# The novel phosphatase NUDT5 is a critical regulator of triple-negative breast cancer growth

**DOI:** 10.1186/s13058-024-01778-w

**Published:** 2024-02-05

**Authors:** Jing Qian, Yanxia Ma, William M. Tahaney, Cassandra L. Moyer, Amanda Lanier, Jamal Hill, Darian Coleman, Negar Koupaei, Susan G. Hilsenbeck, Michelle I. Savage, Brent D. G. Page, Abhijit Mazumdar, Powel H. Brown

**Affiliations:** 1 Department of Clinical Cancer Prevention, The University of Texas MD Anderson Cancer Center, Houston, TX USA; 2 Department of Molecular and Cellular Biology, Baylor College of Medicine, Houston, TX USA; 3 Lester and Sue Smith Breast Center and Dan L. Duncan Cancer Center, Baylor College of Medicine, Houston, TX USA; 4 Present Address: Department of Breast Surgical Oncology, The University of Texas MD Anderson Cancer Center, Houston, TX USA; 5 Faculty of Pharmaceutical Sciences, University of British Columbia, Vancouver, Canada; 6 Present Address: Monte Rosa Therapeutics, Boston, USA; 7 Present Address: Department of Neurosurgery, The University of Texas MD Anderson Cancer Center, Houston, TX, USA; 8 The University of Texas MD Anderson Cancer Center UTHealth Houston Graduate School of Biomedical Sciences, Houston, TX, USA

**Keywords:** Phosphatase, Triple-negative breast cancer, Oxidative stress

## Abstract

**Background:**

The most aggressive form of breast cancer is triple-negative breast cancer (TNBC), which lacks expression of the estrogen receptor (ER) and progesterone receptor (PR), and does not have overexpression of the human epidermal growth factor receptor 2 (HER2). Treatment options for women with TNBC tumors are limited, unlike those with ER-positive tumors that can be treated with hormone therapy, or those with HER2-positive tumors that can be treated with anti-HER2 therapy. Therefore, we have sought to identify novel targeted therapies for TNBC. In this study, we investigated the potential of a novel phosphatase, NUDT5, as a potential therapeutic target for TNBC.

**Methods:**

The mRNA expression levels of NUDT5 in breast cancers were investigated using TCGA and METABRIC (Curtis) datasets. NUDT5 ablation was achieved through siRNA targeting and NUDT5 inhibition with the small molecule inhibitor TH5427. Xenograft TNBC animal models were employed to assess the effect of NUDT5 inhibition on in vivo tumor growth. Proliferation, death, and DNA replication assays were conducted to investigate the cellular biological effects of NUDT5 loss or inhibition. The accumulation of 8-oxo-guanine (8-oxoG) and the induction of γH_2_AX after NUDT5 loss was determined by immunofluorescence staining. The impact of NUDT5 loss on replication fork was assessed by measuring DNA fiber length.

**Results:**

In this study, we demonstrated the significant role of an overexpressed phosphatase, NUDT5, in regulating oxidative DNA damage in TNBCs. Our findings indicate that loss of NUDT5 results in suppressed growth of TNBC both in vitro and in vivo. This growth inhibition is not attributed to cell death, but rather to the suppression of proliferation. The loss or inhibition of NUDT5 led to an increase in the oxidative DNA lesion 8-oxoG, and triggered the DNA damage response in the nucleus. The interference with DNA replication ultimately inhibited proliferation.

**Conclusions:**

NUDT5 plays a crucial role in preventing oxidative DNA damage in TNBC cells. The loss or inhibition of NUDT5 significantly suppresses the growth of TNBCs. These biological and mechanistic studies provide the groundwork for future research and the potential development of NUDT5 inhibitors as a promising therapeutic approach for TNBC patients.

**Supplementary Information:**

The online version contains supplementary material available at 10.1186/s13058-024-01778-w.

## Background

Female breast cancer has emerged as the most commonly diagnosed cancer worldwide, excluding basal cell carcinoma, surpassing lung cancer [1]. In the United States, breast cancer accounts for one out of every three newly diagnosed cancers in women annually [2]. The majority of breast cancer patients bear tumors that express druggable targets: estrogen receptor (ER), progesterone receptor (PR), and human epidermal growth factor receptor 2 (HER2). Patients with ER-positive tumors can benefit from anti-estrogen therapy, which includes selective estrogen receptor (ER) modulators and aromatase inhibitors. For patients with HER2-positive tumors, standard treatments include anti-HER2 therapies, such as anti-HER2 monoclonal antibodies, and anti-HER2 drug conjugates [3]. In contrast, treatment options for patients with triple-negative breast cancers (TNBCs) are very limited, as these tumors lack expression/overexpression of these targetable proteins. While most TNBCs are treated with non-specific chemotherapy, targeted therapies are available for specific subsets of TNBCs. TNBC patients who harbor germline *BRCA1* or *BRCA2* mutations can receive poly (ADP-ribose) polymerase (PARP) inhibitors, and those with TNBC tumors expressing PD-L1 can be treated with immunotherapy [4]. However, the vast majority of TNBC patients do not have effective targeted therapy options available to them. Therefore, our goal is to identify novel targeted therapies for TNBC tumors.

To identify novel targets for the treatment of TNBCs, our previous studies explored kinases and cytokines as potential candidates [5, 6]. Notably, we demonstrated that inhibiting death-associated protein kinase 1 (DAPK1), a regulator of the mTOR pathway, led to the suppression of growth in p53-mutant breast tumors [7]. More recently, we conducted bioinformatic analyses on phosphatases expressed in tumor samples from both TNBC and ER-positive breast cancer patients [8–10]. We identified two distinct clusters of phosphatases in TNBCs: one with 82 overexpressed and another with 64 under-expressed phosphatases when compared to ER-positive tumors [9]. From these studies, we uncovered that one of the overexpressed phosphatases, PTP4A3, promotes TNBC tumor growth [9]. Additionally, we identified under-expressed phosphatases, including PPM1A and DUSP4, as important tumor suppressor genes [10, 11]. In this study, our focus shifted to another overexpressed phosphatase, Nudix (nucleoside diphosphates linked to moiety-X) Hydrolase 5 (NUDT5), which we demonstrated to be a crucial regulator of TNBC proliferation.

NUDT5 (NUDIX5) is a member of the NUDIX hydrolases superfamily [12]. The NUDIX hydrolases function as important enzymes in nucleotide metabolism. NUDT5 is known to hydrolyze two well-characterized substrates, 8-oxo-dGDP and adenosine 5′diphosphoribose (ADPR) [13]. Recent studies have highlighted the significance of NUDT5 in regulating nuclear ATP dynamics and ADPR-related metabolism functions in ER-positive breast cancer cells [14–16]. Additionally, NUDT5 was shown to be associated with the prognosis of breast cancer, lung cancer and prostate cancer [17–19]. Here, we investigated the role of NUDT5 in regulating cell growth and assessing the effects of NUDT5 inhibition on oxidative DNA damage in TNBCs.

Our results demonstrate that NUDT5 exhibits high expression levels in TNBC patients. Loss of NUDT5 inhibits TNBC proliferation and induces growth suppression by causing oxidative DNA damage and interfering with the DNA replication fork. These findings highlight the important biological role of NUDT5 in TNBCs and suggest its potential as a novel target for treating TNBC patients. Understanding the mechanism by which NUDT5 inhibition suppresses TNBC growth lays a solid foundation for the future development of NUDT5 inhibitors as targeted therapies for TNBC patients.

## Materials and methods

### Bioinformatic analysis

The mRNA expression levels of NUDT5 in patient samples were obtained from the TCGA [20] dataset via the Oncomine platform (www.oncomine.org) [21] and the METABRIC [22–24] dataset via the cBioPortal (https://www.cbioportal.org) [24]. NUDT5 mRNA expression levels were reported as Log_2_Median-centered intensity. The differences in NUDT5 expression levels among different subtypes of breast cancer were determined using one-way Analysis of Variance (ANOVA) with Bonferroni's multiple comparisons test. For breast cancer cell lines, NUDT5 mRNA expression levels were acquired from the Cancer Cell Line Encyclopedia [25] and reported as Log_2_ (Fragments per kilobase million). To identify overexpressed phosphatases, we compared TNBC versus ER−/HER2+ using the Bonnefoi [26] dataset, and TNBC versus non-TNBC were obtained from the Bittner data set (Bittner et al*.* International Genomics Consortium (IGC) 2005) through the Oncomine platform [21]. The differences in NUDT5 expression levels between each group were determined by the Student’s *t* test. The survival data of breast cancer patients were obtained from multiple sources: METABRIC [22–24], Esserman [27], Kao [28] and Pawitan [29] datasets via the Oncomine platform (www.oncomine.org) [21], with data retrieved in 2018. The Kaplan–Meier survival curves were generated by dichotomizing patients at the mean expression level of NUDT5. The prognostic impact of NUDT5 expression was determined using Mantel–Cox log-rank analysis.

### Cell line culture

Breast cancer cell lines were obtained from American Type Culture Collection (Manassas, VA). MCF7, MDA-MB-361, ZR-75-1, MDA-MB-231, MDA-MB-436, BT20, T47D, MDA-MB-468, and HEK293T cell lines were passaged and cultured in Dulbecco’s modified Eagle medium (DMEM) and supplemented with 10% regular fetal bovine serum; MCF10A and MCF12A cell lines were cultured in DMEM/F12 medium with 5% horse serum, 20 ng/mL EGF, 0.5 mg/mL hydrocortisone, 100 ng/mL cholera toxin, and 10 μg/mL insulin (Cellgro, Mediatech, Inc., Manassas, VA). HCC1937, HCC70, HCC1143 and BT474 cells were cultured in Roswell Park Memorial Institute medium (RPMI) supplemented with 10% regular fetal bovine serum. Growth media for all cell lines was supplemented with 100 mg/mL penicillin/streptomycin (Gibco™ 15,140,122, Thermo Fisher Scientific, Waltham MA). Cell line identities were confirmed through short tandem repeat DNA fingerprinting, as previously described [10]. A Lonza Mycoplasma Detection Kit (LT07-418; Lonza Walkersville, Inc.,) was used according to manufacturer instructions to detect mycoplasma.

### siRNA transfection

NUDT5-specific targeting siRNA oligos were purchased from Sigma-Aldrich (SASI_Hs01_00109215, SASI_Hs02_00345134, 3’UTR siRNA: 5′ UGA AAG GGC UCU CCA GAU A 3′; St. Louis, MO). Cells were seeded at 50% confluency 1 day prior to siRNA transfection. A mixture of 20 nmol/L siRNA with DharmaFECT1 (T-2001-03; Dharmacon, Lafayette, CO) transfection reagent was added to the cells, with the reagent volume adjusted according to the manufacturer’s recommendations. Non-specific siLuc (SIC001; Sigma-Aldrich) was used as the negative control. Cells were harvested or reseeded for the next analysis after at least 48 h of siRNA transfection.

### Plasmid and plasmid transfection

NUDT5 ORF cDNA plasmid (OHu06714) and vector control (pcDNA3.1+/C-(K)-DYK) were purchased from GenScript (Piscataway, NJ). X-treme-Gene9 transfection reagent (XTG9-RO; Roche) was used for the transfection according to the manufacturer's instructions.

### Western blot and RT-qPCR analyses

Primary antibodies included: NUDT5 antibody (ab129172, 1:1000, Abcam, Cambridge, United Kingdom), and vinculin antibody (V9131, 1:1000, Sigma-Aldrich). Secondary anti-mouse and anti-rabbit horseradish peroxidase antibodies (1:1000) were obtained from GE Healthcare Bio-Sciences Corp (Piscataway, NJ). All of the target proteins and loading controls were processed in parallel. Western blots were performed in triplicate, following previously published methods [10].

RNA was extracted from the cells using RNeasy Mini Kit (74004, Qiagen, Germany). RNA was reverse transcribed into cDNA using random primers, and SuperScript II Reverse Transcriptase (18,064,022, Invitrogen, Waltham MA). The primers and probes for TaqMan-based NUDT5 and Cyclophilin were as follows: NUDT5: Forward primer CTCCGGGAGCTTGAAGAAGA, Reverse primer TTGACAAGCCTGGGTCCATA, Probe TGCCGAATGTTCTCCAGCGGTC (with 5′Fam and 3′Tamra labels), and Cyclophilin: Forward primer ACGGCGAGCCCTTGG, Reverse primer TTTCTGCTGTCTTTGGGACCT, Probe CGCGTCTCCTTTGAGCTGTTTGCA. Real-time quantitative PCR was performed using the QuantStudio 7Pro system (Applied Biosystems, Waltham, MA). Cyclophilin served as the endogenous control, and the relative gene expression was determined through the 2 ^− ΔΔCt^ method.

### Cell growth assays

Cells were treated either with siLuc control or siNUDT5 for 48 h to induce knockdown. Subsequently, cell numbers were counted using a Countess automated cell counter (Invitrogen, Waltham MA) and reseeded at a density of 1000 cells per well in 96-well plates. These cells were cultured in the indicated media for a period of 7 days. For cell growth analyses, 4′,6-diamidino-2-phenylindole (DAPI)–stained cell nuclei were imaged at days 1, 3, 5, and 7 using an ImageXpress Pico microscope (Molecular Devices, San Jose, CA) and analyzed using CellReporterXpress image acquisition and analysis software. Cells were treated with either DMSO or 10 μM TH5427 on day 1 and Hoechst-stained (20 µM, Thermo Fisher Scientific, Hoechst 33342 Solution, Waltham MA) cell nuclei were imaged at days 1, 3, 5, and 7. The half maximal inhibitory concentration (IC_50_) of TH5427 was calculated using the 4 parameter logistic regression models by Prism 9.1 (GraphPad). The cells were individually plated into each well as quadruplicates, and cell numbers were reported as average cell count ± SD.

### Xenograft growth

This study was conducted following animal protocols approved by The University of Texas M.D. Anderson Cancer Center Institutional Animal Care and Use Committee (IACUC). Female nude mice (The Jackson Laboratory, Bar Harbor ME) aged 4–6 weeks, were used in the experiments. TH5427 (Cat. No. 6534) compound was purchased from TOCRIS (Bristol, United Kingdom). One million MDA-MB-231 cells were subcutaneously injected into the 2nd mammary fat pad of 20 nude mice. When tumor size reached 50 mm^3^, these 20 mice were randomly divided into two groups of 10 each. One group of mice received intraperitoneal (i.p.) injections of vehicle (water), while the other group received i.p. injections of TH5427 (50 mg/kg) 5 times per week. Xenograft tumors were measured twice per week, and tumor volumes were calculated using the formula V = 0.5(width^2^ × length). Individual tumor growth rates were calculated by log-transformed linear regression slopes. Mice were sacrificed when the largest tumor reached 1000 mm^3^. 4 mice in the TH5427 treatment group were found dead after day 7. Growth rates were compared between the slopes of the vehicle and treatment groups using a Student’s *t*-test.

### H&E and immunohistochemistry

Tumor samples from the mice were processed by fixation in a 1:10 formalin solution and subsequently embedded in paraffin. Hematoxylin–eosin staining (H&E) and immunohistochemical staining (IHC) of tumor tissue slides was performed, as previously described [22]. For H&E and IHC staining, 3 slides from each treatment group were used. For IHC, samples were incubated with Ki67 primary antibody (Lab Vision, 1:1000) or NUDT5 primary antibody (ab129172, Abcam, 1:500). Slides were processed at the Baylor College of Medicine Breast Center Pathology Core. Cell pellet blocks of MDA-MB-361 and MDA-MB-231 cells were used as negative and positive controls for the NUDT5 primary antibody. The images were acquired using Aperio ImageScope (Leica Biosystems, Illinois US) and processed with Aperio ImageScope Pathology Slide Viewing Software (Leica Biosystems, Illinois US).

### Proliferation, death, apoptosis, and ROS assays

Cells were seeded into 96-well plates at a density of 5000 cells per well. To synchronize the cell cycle, cells were incubated with Lovastatin (10 μM) for 48 h, then incubated with 1 mM mevalonate for 24 h to release them back to the normal cell cycle. Proliferation was assayed by bromodeoxyuridine (BrdU) incorporation using the Roche Cell Proliferation ELISA BrdU chemiluminescent kit, according to the manufacturer's instructions. Dying cells were detected by DRAQ7™ (3 µM, Abcam, ab109202) positivity and nuclei were counterstained with Hoechst (20 µM, Thermo Fisher Scientific™, Hoechst 33342 Solution, Waltham MA). Images were captured via ImageXpress® Pico microscope (Molecular Devices, San Jose, CA) and analyzed with CellReporterXpress image acquisition and analysis software. Apoptosis was detected by Annexin V-PI positivity (Invitrogen, Annexin V-FITC Conjugates, Waltham MA). Flow cytometry analysis was conducted with the assistance of the Flow Cytometry and Cellular Imaging Core Facility North Campus at The University of Texas MD Anderson Cancer Center. Experimental data points were collected in biological triplicates, and results were reported as average ± SD. We measured the reactive oxygen species (ROS) level of breast cancer cells using the ROS-Glo™ H_2_O_2_ Assay kit (Promega, Madison MI) according to manufacturer’s protocol. The assay is a two-reagent-addition protocol. The H_2_O_2_ substrate (provided by the kit) reacts directly with H_2_O_2_ to create the luciferin precursor, which then is converted to luciferin and reacts with luciferase (provided by the kit) to generate a luminescent signal, so that the signal is proportional to the H_2_O_2_ level. The luminescence was recorded by a BioTek Synergy Mx Microplate Reader (BioTek, Winooski, VT). The measurement of breast cancer cells was conducted both at basal level and after oxidative induction with 50 μM H_2_O_2_ for 6 h.

### Immunofluorescence staining

After 72 h of siRNA treatment, cells were seeded at a density of 5000 cells per well in 96-well plates. Cell fixation was performed with cold methanol after 4 days for 8-oxoG staining and 7 days for γH_2_AX staining. Samples were incubated with 8-oxoG (Santa Cruz Biotechnology, sc-130914, 1:50) and γH_2_AX (Cell Signaling Technology, #9718, 1:1000) antibodies at 4 °C overnight. The following day, samples were incubated with the secondary antibodies Alexa Fluor 488 (Invitrogen, A28175, 1:1000) and Alexa Fluor 594 (Invitrogen, A-11032, 1:1000), accordingly, for 1 h at room temperature. The images were obtained using the ImageXpress® Pico microscope (Molecular Devices, San Jose, CA) and analyzed with CellReporterXpress image acquisition and analysis software. Experimental data points were performed in quadruplicate, and results were reported as average ± SD. 8-oxoG intensity and γH_2_AX-positivity were compared between siLuc and siNUDT5 using the Student's *t*-test.

### DNA fiber assay

After 48 h of siRNA treatment, 10,000 MDA-MB-231 and MCF7 cells were seeded into 6-well plates. The following day, cells were exposed to 50 μM 5-Iodo-2'-Deoxyuridine (IdU) for 30 min, followed by a wash with phosphate-buffered saline, and treated with 100 μM 5-chloro-2′-deoxyuridine (CIdU) for another 30 min. The DNA fiber assay was performed as described [30]. First, samples were incubated with a primary antibody mix of rat monoclonal anti-BrdU antibody (Abcam, ab6326, 1:500) and mouse monoclonal anti-BrdU antibody (BD Biosciences, 347580, 1:500, San Jose, CA) in a blocking solution overnight at 4 °C. Samples were then incubated with a secondary antibody mix composed of Alexa Fluor 594 goat anti-rat IgG (1:1000, ThermoFisher Scientific, A-11007) and Alexa Fluor 488 goat anti-mouse IgG (1:1000, ThermoFisher Scientific, A-11001). DNA fibers were imaged with Andor Revolution XDi WD Spinning disk confocal microscope (Oxford Instruments plc, UK) with assistance from the University of Texas MD Anderson Cancer Center North Campus Flow Cytometry and Cellular Imaging Core Facility. The length of the individual fibers (up to 40 fibers in each group) were measured using the microscopy image analysis software Imaris (Oxford Instruments plc, UK). Statistical differences of the mean fiber length were compared using a Student’s *t* test and reported as average ± SD.

### Statistics

All graphs were presented as mean ± standard deviation. Two-tailed Student’s *t* tests were used to determine the statistical significance between two different groups, and one-way ANOVA tests were used to determine the statistical significance among multiple groups. *p*-values less than 0.05 were considered statistically significant,, with symbols used to denote levels of significance as follows: * represents *p* ≤ 0.05, ** represents *p* ≤ 0.01, *** represents *p* ≤ 0.001, **** represents *p* ≤ 0.0001.

## Results

### NUDT5 is highly expressed in TNBC

To identify highly expressed phosphatases that are specific to TNBCs, we previously performed a microarray RNA profiling study involving 332 phosphatase genes, comparing human ER-negative tumor samples to ER-positive tumor samples. By analyzing 102 breast cancers, we identified a subset of 82 overexpressed phosphatases and 64 underexpressed phosphatases in TNBC in comparison to ER-positive breast cancers [9]. For this study, we integrated our previous microarray analysis of overexpressed phosphatases with data from three other publicly available datasets. Specifically, we compared gene expression in (1) TNBC tumor tissues to normal tissues [22], (2) TNBC tumors to non-TNBC tumors (Bittner et al. International Genomics Consortium (IGC) 2005), and (3) TNBC tumors to ER-negative/HER2-positive tumors [26]. In this integrative analysis, we observed overexpression of CDC25A, CDC25B, DLGAP5, IMPA2, NUDT5, and PTPLA across four different datasets (Additional file 1: Figure S1). Among these six identified phosphatases, NUDT5 consistently exhibited an association with breast cancer outcomes, with high NUDT5 expression correlating with poor outcomes (Additional file 9: Table S1). As a result, for this study we focused on the biologic function and mechanistic role of NUDT5 in TNBC.

**Fig. 1:**
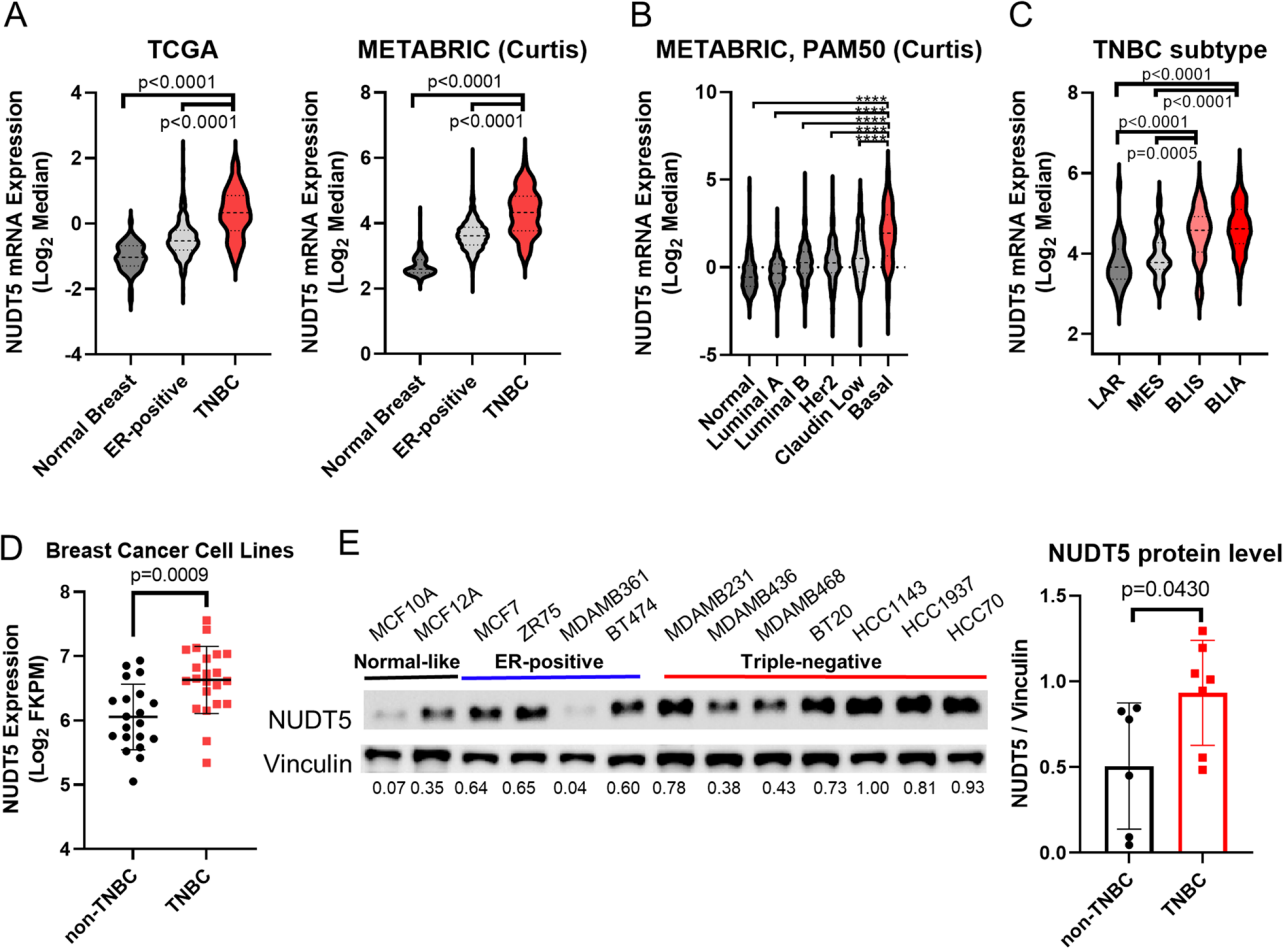
NUDT5 expression in breast cancer. **A** NUDT5 mRNA expression levels from publicly available databases (from The Cancer Genome Atlas and Molecular Taxonomy of Breast Cancer International Consortium [METABRIC]). The mRNA expression level of NUDT5 is compared between TNBC and ER-positive breast cancers, as well as TNBC and normal breast. **B** NUDT5 mRNA expression levels across the different PAM50 subgroups using the METABRIC data. The mRNA expression level of NUDT5 is compared between basal subtype and the normal-like, Luminal A, Luminal B, HER2-enriched, and Claudin-low subtypes. **C** NUDT5 expression across the different triple-negative breast cancer (TNBC) subtypes (from Burstein et al. [32]) using data from METABRIC. **D** NUDT5 mRNA expression in non-TNBC and TNBC cell lines using data from the Cancer Cell Line Encyclopedia is shown [25]. **E** NUDT5 protein expression is shown by Western blot analysis of multiple cell lines, including normal-like, estrogen receptor-positive, and TNBC cell lines. Quantification of NUDT5 protein levels in non-TNBC and TNBC cells is also shown. The differences of NUDT5 expression levels between two subtypes were determined by Student’s *t *test and the differences among multiple subtypes were determined by one-way ANOVA. (*ns* not significant; *p < 0.05; **p < 0.01; ***p < 0.001; ****p < 0.0001)

We initiated our analysis by examining NUDT5 mRNA expression in the METABRIC [22, 23] and TCGA [20, 31] datasets, two different, independent publicly-available datasets. Our observations revealed that NUDT5 mRNA levels are significantly higher in TNBCs than in ER-positive tumors and normal breast tissue samples (Fig. 1A). To delve further into the expression patterns, we stratified NUDT5 RNA expression levels in the METABRIC dataset across the PAM50 breast cancer subtypes (normal-like, luminal A, luminal B, HER2-enriched, claudin-low, and basal-like). Notably, NUDT5 exhibited its highest expression in basal-like subtype tumors, which are predominantly TNBCs (Fig. 1B). For a more refined investigation of NUDT5 expression within the various subtypes of TNBC (luminal androgen receptor (LAR), mesenchymal (MES), basal-like immune-activated (BLIA), basal-like immunosuppressed (BLIS) [32]) using the METABRIC [22, 23] dataset, our findings demonstrated that NUDT5 is most highly expressed in the BLIS and BLIA TNBC subtypes (Fig. 1C). Utilizing RNA-seq data from the Cancer Cell Line Encyclopedia [25], we found that TNBC cell lines express more NUDT5 mRNA than non-TNBC cell lines (Fig. 1D). To confirm that the observed elevated NUDT5 RNA expression correlates with increased NUDT5 protein expression, we performed a Western-blot analysis of breast cancer cells (two normal-like breast cell lines: MCF-10A and MCF-12A; four ER-positive breast cancer cell lines: MCF-7, ZR-75-1, MDA-MB-361 and BT-474; and seven TNBC cell lines: MDA-MB-231, MDA-MB-436, MDA-MB-468, BT-20, HCC1143, HCC1937 and HCC70), all of which were in the exponential phase in cell culture. Our results demonstrated that NUDT5 protein is more abundant in the TNBC cell lines than in the non-TNBC cell lines (Fig. 1E).

### NUDT5 loss inhibits TNBC growth

**Fig. 2:**
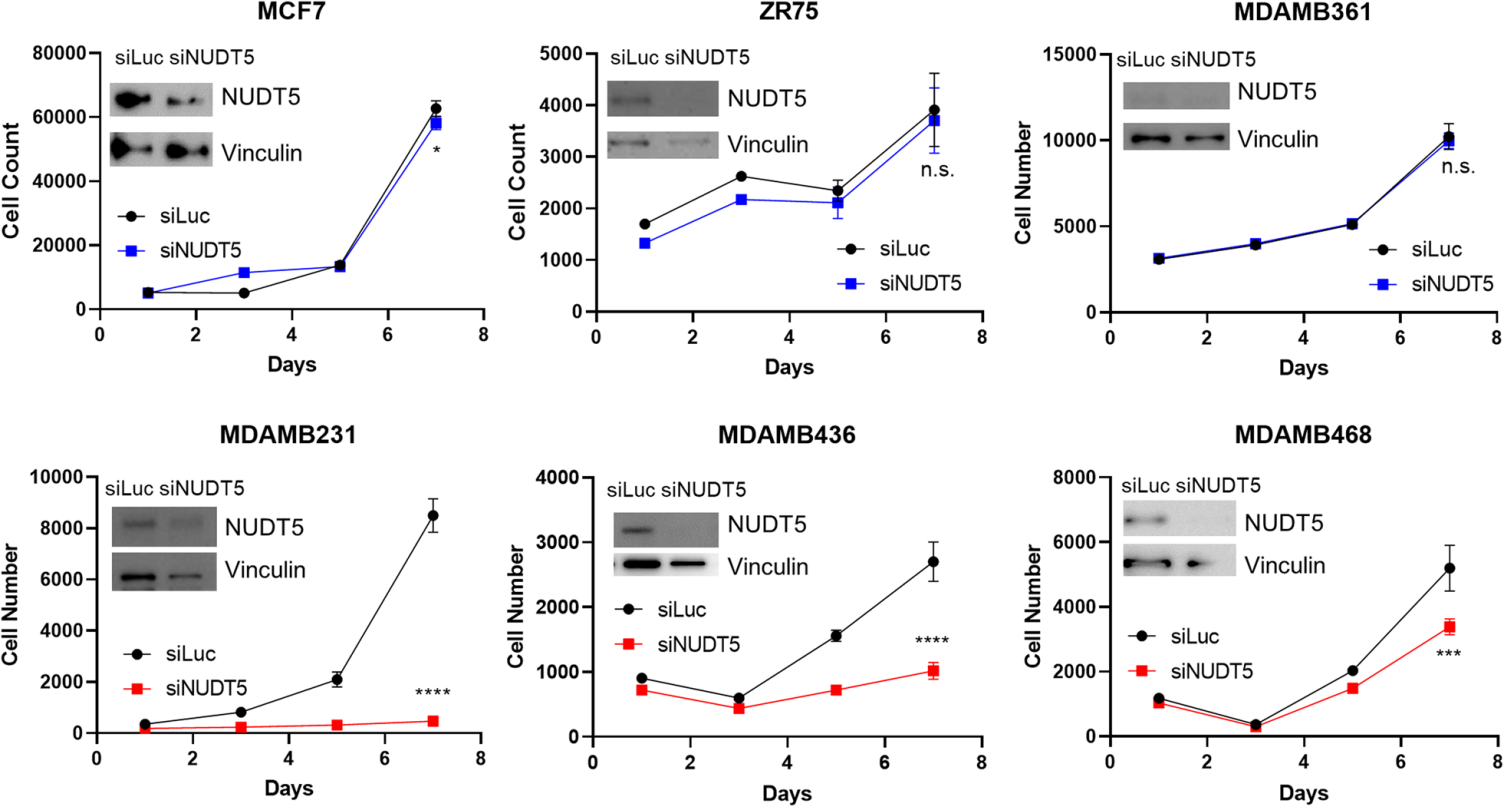
NUDT5 loss inhibits triple-negative breast cancer growth. **A** Cell growth of estrogen receptor-positive breast cancer cells (MCF7, ZR-75-1 and MDA-MB-361), and triple-negative breast cancer cells (MDA-MB-231, MDA-MB-436, and MDA-MB-468) following siLuc or siNUDT5 treatment. Knockdown efficiency of protein samples harvested at Day 7 is shown by Western blot analysis (protein expression at D1 and D7 is also shown in Additional file 2: Figure S2A). RNA expression after siRNA knockdown at D1 and D7 is also shown in Additional file 2: Figure S2A. The significant differences between day 7 cell counts were determined using Student’s *t* test (*ns* not significant; *p < 0.05; **p < 0.01; ***p < 0.001; ****p < 0.0001). Shown in this figure is one representative growth experiment for each cell line. This experiment was repeated in each of these cell lines with similar results showing effective siRNA knockdown (at the RNA and protein levels), as well as effective growth suppression in the TNBC cell lines

To investigate the impact of NUDT5 on the growth of breast cancer cells, we employed siRNA-mediated knockdown in a panel of breast cell lines, including 3 ER-positive cell lines (MCF-7, ZR-75-1 and MDA-MB-361) and 3 TNBC cell lines (MDA-MB-231, MDA-MB-436 and MDA-MB-468). The efficacy of siRNA knockdown was demonstrated by both Western-blot and quantitative polymerase chain reaction (qPCR), as shown in Additional file 2: Figure S2A. As depicted in Fig. 2A, the depletion of NUDT5 had no effect on the growth of the ER-positive cell lines ZR-75-1 and MDA-MB-361, with a modest reduction observed in the MCF-7 cell line. In contrast, NUDT5 loss significantly suppressed the growth of the TNBC cell lines MDA-MB-231, MDA-MB-436 and MDA-MB-468. Therefore, siRNA-mediated NUDT5 depletion had a minimal impact on the growth of ER-positive breast cancer cells, and a more profound effect on TNBC cells. To confirm the specificity of siRNA-mediated knockdown, we overexpressed a NUDT5 cDNA in TNBC cells treated with siRNA targeting the 3’UTR region of NUDT5 mRNA. The overexpression of the siRNA-resistant NUDT5 successfully restored the growth of MDA-MB-436, providing further evidence for the importance of NUDT5 in the growth of TNBC (Additional file 2: Figure S2B).

### NUDT5 small molecule inhibitor TH5427 suppresses TNBC growth in vitro and in vivo

**Fig. 3:**
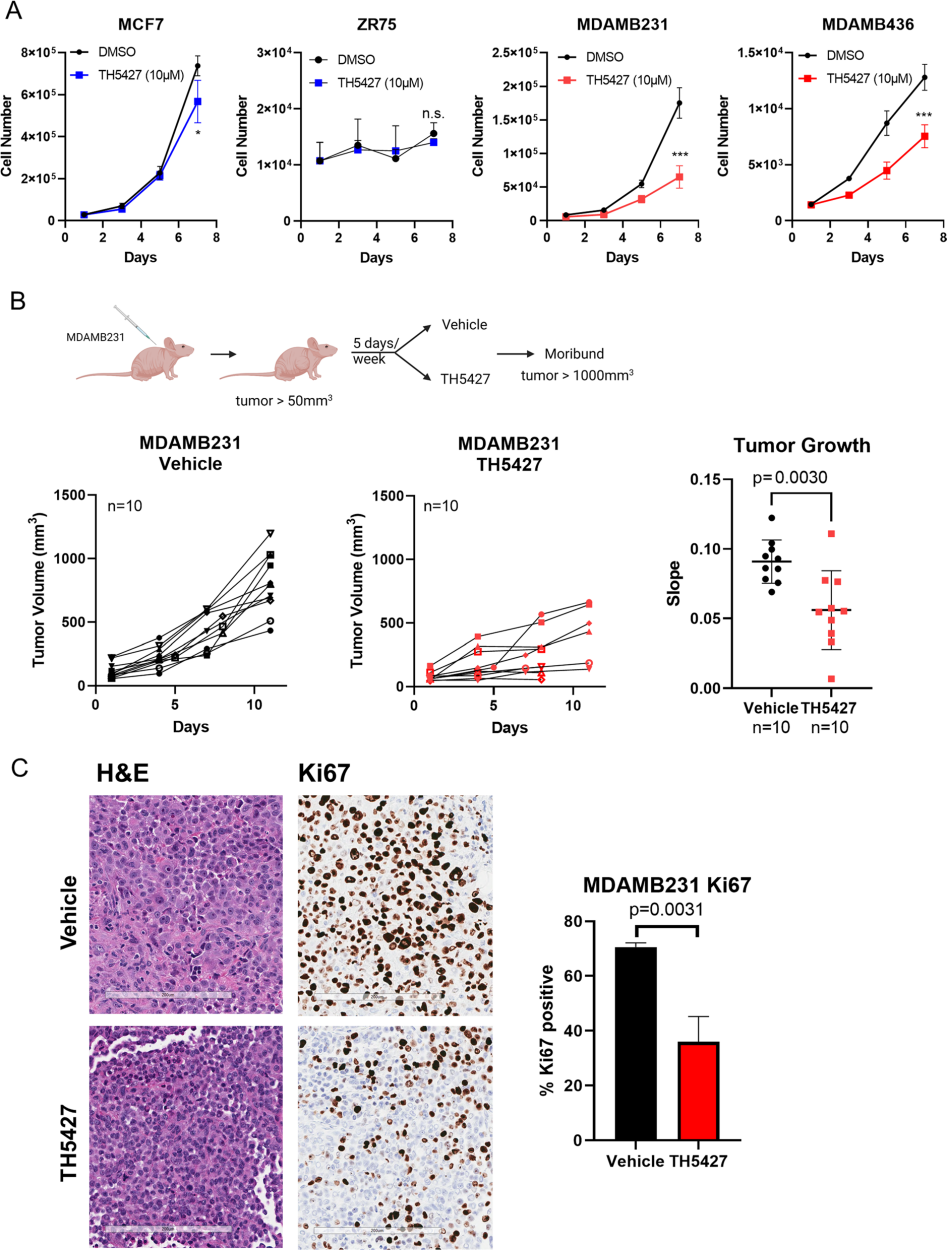
NUDT5 small molecule inhibitor TH5427 suppresses triple-negative breast cancer growth in vitro and in vivo. **A** ER-positive cell lines MCF7 and ZR-75-1 and TNBC cell lines MDA-MB-231 and MDA-MB-436 were treated with DMSO control or 10 μM TH5427 at day 1. Cell counts were recorded on days 1, 3, 5, and 7. **B** Schematic illustration of MDA-MB-231 xenograft tumors. 20 Nude mice were injected with MDA-MB-231 cells, and randomized into two groups of 10 nude mice each, which were treated with either vehicle or 50 mg/kg TH5427 via intraperitoneal injection 5 times per week. Mice were sacrificed when the largest tumor size reached 1000 mm^3^. 4 mice were found dead after 7 days of TH5427 treatment. Tumor volumes were calculated using the formula (width × width × length)/2. Tumor growth was analyzed using linear regression of log_10_ (tumor volume), and the difference between tumor growth slopes was compared by Student’s *t* test (*p < 0.05; ***p < 0.001). **C** H&E and IHC staining of tumor samples from 20 female nude mice injected with MDA-MB-231 cells into the mammary fat pad. H&E and IHC images of one representative tumor are shown. Additional images from 4 other tumors (2 tumors from vehicle-treated mice and 2 tumors from TH5427-treated mice) are presented in Additional file 3: Figure S3C. Ki67 positivity was compared between treatment and control groups. Also see Additional file 3: Figure S3D of the NUDT5 expression via IHC analysis of the 6 independent tumors outlined in **3C** above (3 tumors from vehicle-treated mice and 3 tumors from TH55427-treated mice). NUDT5 expression is also shown via IHC analysis of MDA-MD-361 cells (negative control) and MDA-MB-231 cells (positive control). The statistical difference in these analyses was determined by Student’s *t* test

TH5427, a potent small molecule inhibitor targeting NUDT5, was previously identified by Page et al. [33]. To assess the impact of TH5427 in vitro, we treated the ER-positive cell lines MCF-7 and ZR-75-1, as well as the TNBC cell lines MDA-MB-231 and MDA-MB-436, with 10 µM TH5427 or dimethyl sulfoxide (DMSO) as a control on day 1. We then measured cell growth over a 7-day period by cell counting. As illustrated in Fig. 3A, TH5427 significantly suppressed the growth of TNBC cells, while it only marginally inhibited the growth of the ER-positive cells. Additionally, we conducted dose–response studies of TH5427 across various breast cell lines (Additional file 3: Figure S3A). The half maximal inhibitory concentrations (IC_50_) of TH5427 in the TNBC cell lines (MDA-MB-231, MDA-MB-436, MDA-MB-468 and BT-20) were found to be significantly lower than those in the ER-positive cell lines (MCF-7, MDA-MB-361, T-47D and ZR-75–1) and normal-like breast cell lines (MCF-10A and MCF-12A; Additional file 3: Figure S3B).

In our next step, we sought to assess the anti-tumor efficacy of TH5427 in vivo. To conduct these experiments, we injected MDA-MB-231 cells into the mammary fat pad of 20 female nude mice. Upon reaching a tumor volume of 50 mm^3^, we randomly assigned the mice into two groups, with each group consisting of 10 mice. One group received intraperitoneal injections of vehicle control, while the other group received TH5427 (50 mg/kg) through the same route, administered 5 days a week. This regimen continued until the largest tumor reached 1000 mm^3^. 4 mice were found dead after 7 days in the TH5427 treatment group. As illustrated in Fig. 3B, the tumors in mice treated with TH5427 exhibited slower growth compared to those in the vehicle-treated mice. To assess this growth reduction, we performed statistical analysis by calculating the growth rate for each individual tumor. This involved conducting linear regression on log-transformed growth curves for each tumor and determining the slope of these curves. Subsequently, we compared the mean slopes between the two treatment groups using the Student’s *t* test. This analysis demonstrated that TH5427 led to a significant decrease in the tumor growth rate. To gain insights into the effect of TH5427 on the proliferative state of the cells, we collected tumor samples and conducted immunohistochemical analyses. To determine the effect of TH5427 on the cell proliferation state, we performed an immunohistochemical analysis of the tumor samples. Our findings revealed that tumors treated with the NUDT5 inhibitor displayed reduced Ki67 staining, indicative of slowed proliferation (as shown in Fig. 3C and Additional file 3: Figure S3C). It is noteworthy that the NUDT5 levels remained unaltered following TH5427 treatment, as indicated by NUDT5 immunohistochemistry (Additional file 3: Figure S3D).

### NUDT5 depletion leads to proliferation suppression

**Fig. 4:**
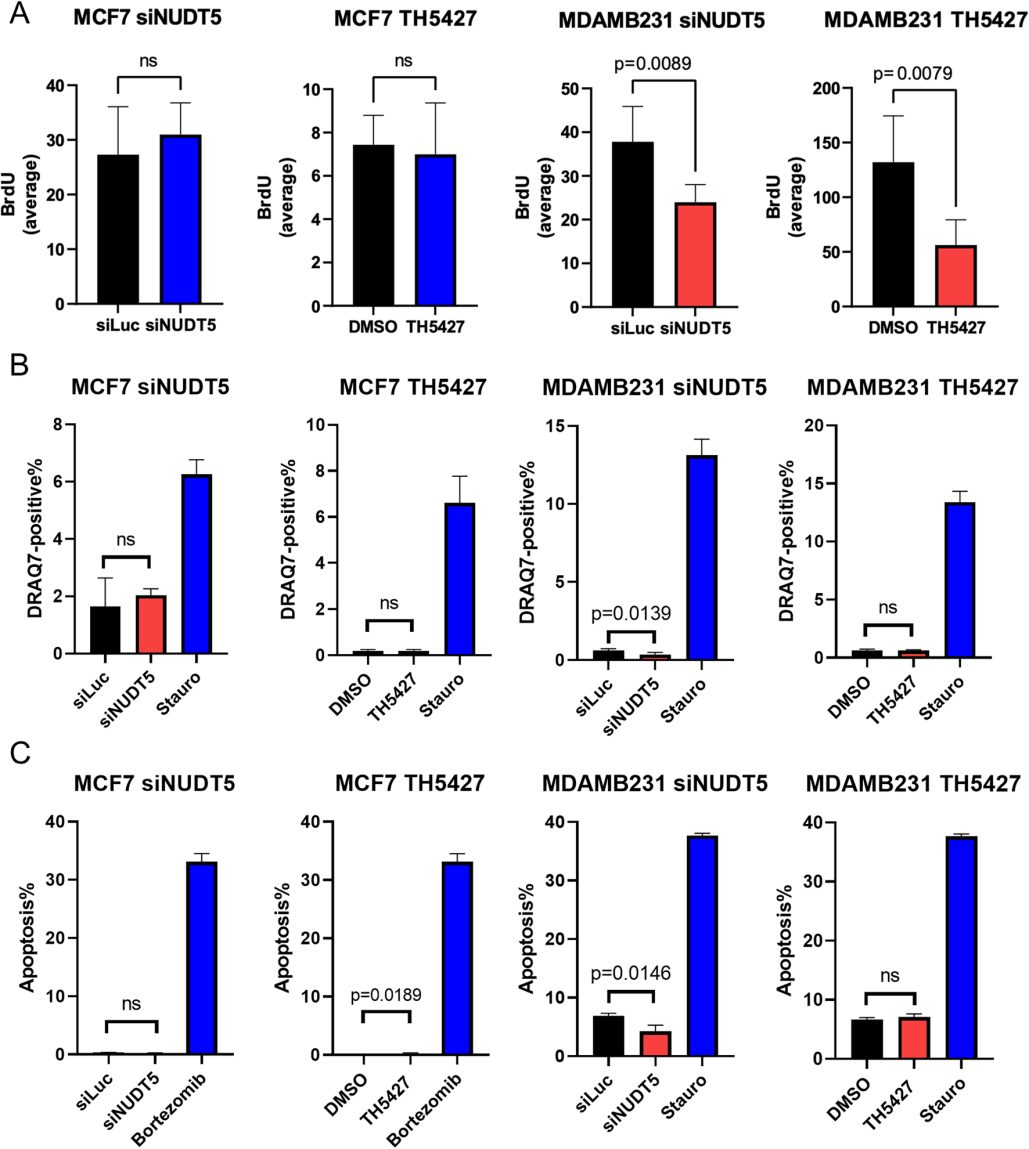
NUDT5 depletion suppresses TNBC cell proliferation. **A** BrdU incorporation in the ER-positive cell line MCF-7 and TNBC cell line MDA-MB-231 treated with siLuc versus siNUDT5, or DMSO versus TH5427. Cells were treated 48 h prior to the assay. **B** DRAQ7 cell death assay in the ER-positive cell line MCF-7 and TNBC cell line MDA-MB-231 treated with siLuc versus siNUDT5, or DMSO versus TH5427. Cells were treated 48 h prior to the assay. 10 µM staurosporine was used as a positive control. **C** Annexin V/ propidium iodide (PI) staining in the ER-positive cell line MCF-7 and TNBC cell line MDA-MB-231 treated with siLuc versus siNUDT5, or DMSO versus TH5427. Cells were treated 48 h prior to the assay. 1 µM bortezomib or 10 µM staurosporine were used as a positive control. The apoptotic cell population is composed of both early (Annexin V-positive/PI-negative) and late apoptotic (Annexin V-positive/PI-positive) cells. Statistical comparisons were analyzed by the Student’s *t *test, and *p *values are shown

To gain insights into the biological mechanism behind the growth suppression observed in TNBC following NUDT5 inhibition, we conducted a series of assays to evaluate cell proliferation, cell death, and apoptosis. Our findings revealed that the inhibition of NUDT5, achieved either through siRNA-mediated knockdown or TH5427 treatment, significantly suppressed BrdU incorporation in TNBC MDA-MB-231 cells. In contrast, this inhibition did not affect BrdU incorporation in ER-positive MCF7 cells (Fig. 4A). However, it's noteworthy that in both the ER-positive MCF7 cell line and the TNBC MDA-MB-231 cell line, siRNA-mediated NUDT5 knockdown or inhibition of NUDT5 using TH5427 did not induce cell death, as evidenced by the absence of positive staining for DRAQ7 (Fig. 4B). Additionally, apoptosis, assessed through Annexin V-PI staining, remained unaffected by NUDT5 inhibition in both cell lines (Fig. 4C, Additional files 4, 5: Figures S4 and S5). Therefore, our results suggest that in TNBC cells, the inhibition or loss of NUDT5 leads to a reduction in proliferation without affecting cell viability.

### Loss of NUDT5 induces oxidative DNA damage and impairs replication fork function

**Fig. 5:**
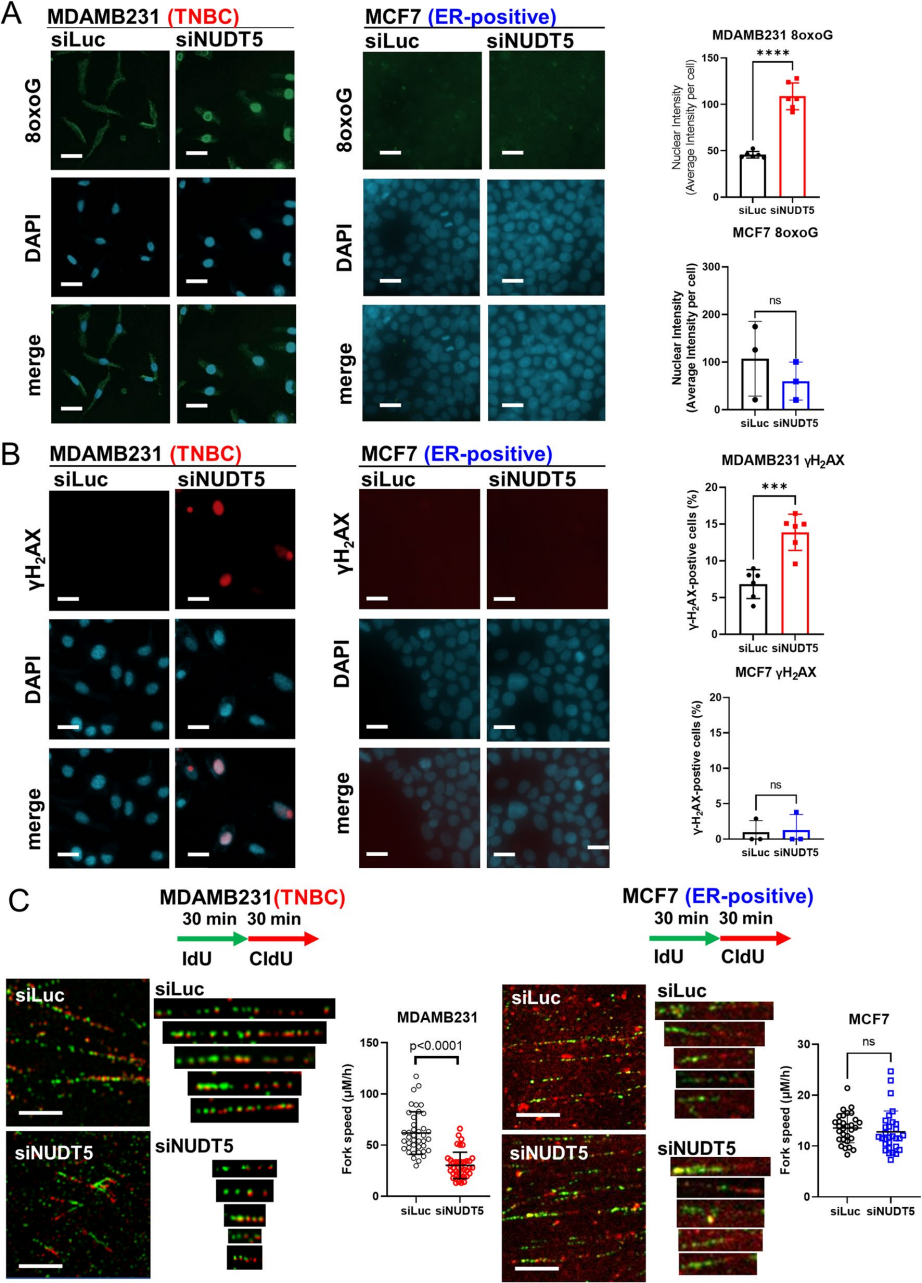
Loss of NUDT5 induces oxidative 8-oxoG and DNA damage response. **A** 8-oxoG lesions were stained in TNBC (MDA-MB-231) and ER-positive (MCF-7) cells treated with siLuc or siNUDT5, and nuclei were counterstained with DAPI after 4 days. The data is shown as nuclear intensity for siLuc- or siNUDT5-treated cells. **B** γH_2_AX was stained in MDA-MB-231 and MCF-7 cells treated with siLuc or siNUDT5, and nuclei were counterstained with DAPI after 7 days. The data is shown as γH_2_AX positivity, and was compared between the different treatments. Statistical significance was analyzed by the Student’s *t *test. **C** Representative DNA fibers from MDA-MB-231 and MCF-7 cells treated with siLuc or siNUDT5. Cells were first labelled with 30 min treatment with 50 µM IdU, followed by 30 min treatment with 100 µM CIdU. DNA fiber length was imaged by an Andor Revolution XDi WD Spinning disk confocal microscope and analyzed with Imaris software. The fork speed = (the length of IdU labeled DNA fiber + the length of CdU labeled DNA fiber)/total labeling time (1 h). The difference between siLuc- and siNDUT5-treated DNA fibers is shown graphically and compared using a Student’s *t* test. Additional TNBC (MDA-MB-436, MDA-MB-468) and ER-positive (ZR-75-1 and MDA-MB-361) cell lines are shown in Additional file 7, 8: Figures S7 and S8. Proof of effective knockdown is shown via Western blot and qPCR in Additional file 2: Figure S2A

It is known that TNBCs exhibit elevated levels of reactive oxygen species (ROS) in comparison to ER-positive cancers [34]. High levels of ROS can lead to the accumulation of 8-oxo-dGDP, a substrate of NUDT5. When phosphorylated, this substrate is known to further induce oxidative DNA damage [13]. To confirm these previously established findings, we compared ROS levels in 4 different breast cancer cell lines. The ROS level is quantified by measuring hydrogen peroxide (H_2_O_2_) using the ROS-Glo™ H_2_O_2_ Assay. We measured both the ROS level at the basal state of proliferating cells and the induced ROS level following hydrogen peroxide induction in 2 ER-positive breast cancer cell lines (ZR-75-1 and MDA-MB-361)) and 2 TNBC cell lines (MDA-MB-231 and MDA-MB-468). Our results affirmed that TNBC cells exhibit a significant accumulation of ROS as compared to non-TNBC cells (Additional file 6: Figure S6). Employing the widely recognized marker for oxidative DNA stress, 8-oxo-guanine (8-oxoG), which is a downstream product of phosphorylated 8-oxo-dGDP, we observed a substantial increase in nuclear 8-oxoG levels after NUDT5 knockdown in TNBC cell lines (MDA-MB-231, MDA-MB-436 and MDA-MB-468) compared to ER-positive breast cancer cell lines (MDA-MB-231 and MCF7 in Fig. 5A; MDA-MB-436, MDA-MB-468, ZR-75-1 and MDA-MB-361 in Additional file 7: Figure S7A, S7B). Additionally, we explored whether the loss of NUDT5 activated the DNA damage response, using γH_2_AX staining. We observed an elevation in γH_2_AX positivity in TNBCs following NUDT5 knockdown (Fig. 5B: MDA-MB-231 and MCF-7 cells; and Additional files 8: Figures S8A, S8B: MDA-MB-436, MDA-MB-468, ZR-75-1 and MDA-MB-361 cells). Taken together, these results show that NUDT5 ablation leads to increased oxidative DNA damage.

To clarify the impact of NUDT5 loss on DNA replication and provide a more comprehensive understanding of how NUDT5 knockdown impedes TNBC cell proliferation, we conducted DNA fiber assays to measure DNA replication fork progression. In this assay, MDA-MB-231 (TNBC) and MCF-7 (ER-positive) cells were treated with either siLuc or siNDUT5 before incorporating IdU and CIdU. By comparing the fiber length between siLuc-treated and siNUDT5-treated samples, we observed that DNA fibers in NUDT5 knockdown cells were notably shorter than those in control cells within the context of TNBC cells. This observation demonstrates that DNA replication is slowed down upon NUDT5 depletion in TNBC (Fig. 5C).

## Discussion

In this study, we studied the overexpressed phosphatase NUDT5 and its impact on the growth and survival of TNBC. Our findings reveal that NUDT5 loss or inhibition leads to the suppression of TNBC cell line growth, whereas it has minimal effect on the growth of ER-positive cell lines. Furthermore, we demonstrated the potent tumor growth inhibitory properties of the NUDT5 small molecule inhibitor, TH5427, both in vitro and in vivo. To uncover the underlying mechanisms responsible for the growth suppression observed upon NUDT5 loss, we conducted a series of cell biological assays to assess cell death, apoptosis, and proliferation. Our results demonstrate that NUDT5 inhibition or loss does not induce cell death, but significantly hinders the proliferation of TNBC cells. Furthermore, NUDT5 loss triggers indicators of oxidative DNA stress, including increased 8-oxoG incorporation and γH_2_AX positivity. Additionally, our investigations show that the depletion of NUDT5 results in a deceleration of DNA replication in TNBC cells, leading to subsequent proliferation suppression and growth inhibition. These findings shed light on the critical role of NUDT5 in regulating the growth of TNBC cells and the potential therapeutic implications of targeting NUDT5 in the treatment of these aggressive breast cancers.

**Fig. 6:**
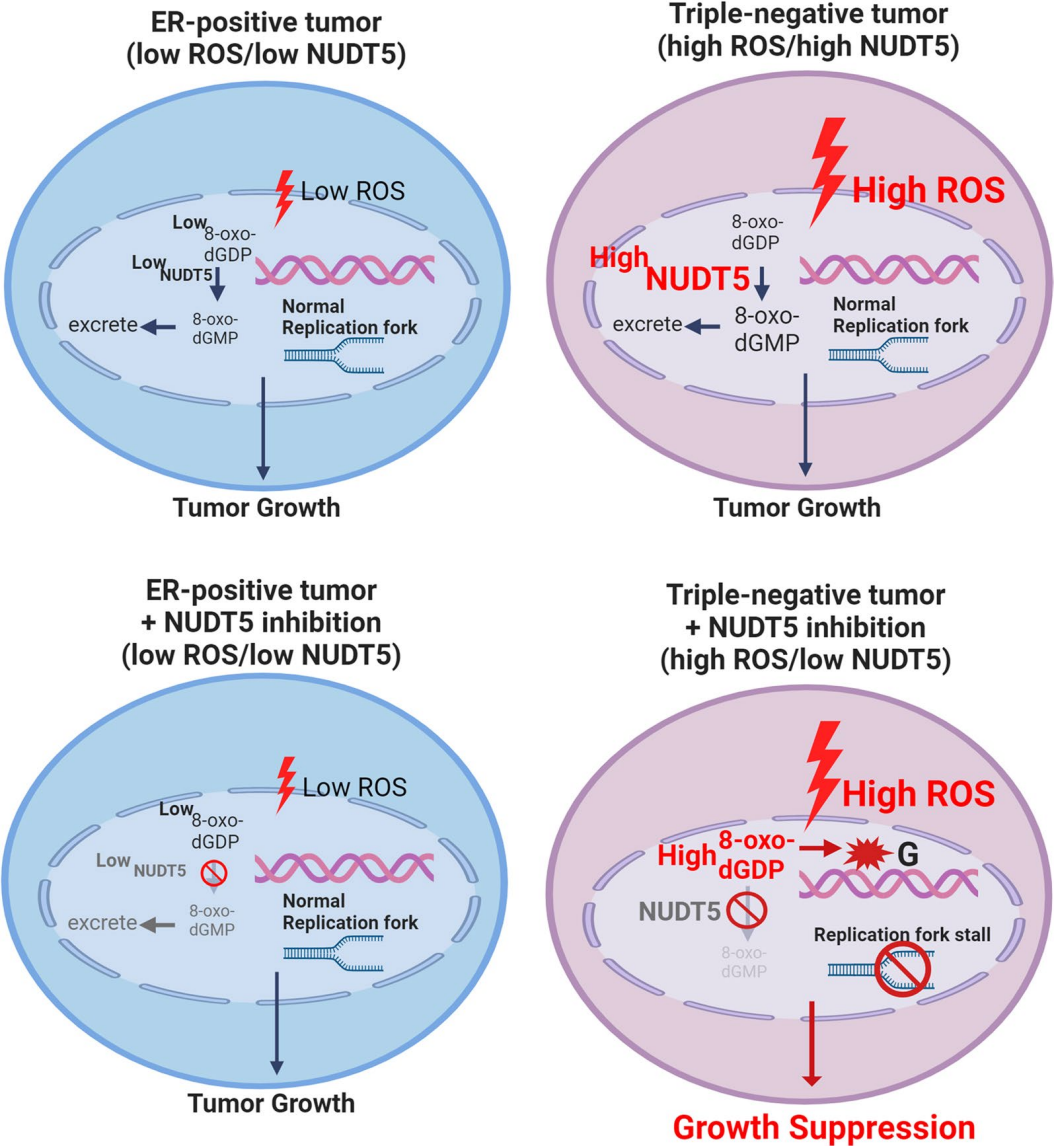
Proposed mechanism of NUDT5 biological function in breast cancers. The level of ROS is low in ER-positive tumors, resulting in low accumulation of 8-oxoG and γH_2_AX lesions in the nucleus. In such cases, the inhibition or loss of NUDT5 does not affect the growth of these ER-positive tumors. In TNBC tumors, both ROS and NUDT5 levels are elevated. When NUDT5 is abundant, it mitigates oxidative DNA damage by hydrolyzing oxidized deoxyribonucleoside diphosphates. Consequently, there is no incorporation of 8-oxoG into the DNA, and γH_2_AX lesions do not accumulate. However, in the absence of NUDT5, uncontrolled oxidative stress on the nucleotide pool occurs. This leads to the incorporation of 8-oxoG lesions into the DNA and the accumulation of γH_2_AX lesions in the nucleus, ultimately causing DNA replication fork slowing and reduced proliferation

The proposed mechanism delineating how NUDT5 regulates TNBC growth is depicted in Fig. 6. In ER-positive tumors, where ROS levels are low and NUDT5 expression is also low, there is minimal accumulation of 8-oxoG or γH_2_AX lesions within the nucleus. Consequently, the loss or inhibition of NUDT5 in this context does not have a substantial impact on cell proliferation. However, in TNBC tumors, both ROS and NUDT5 are present at high levels. In this scenario, the elevated NUDT5 activity counteracts the effects of high ROS, leading to a reduction in the incorporation of 8-oxoG into the DNA and preventing the accumulation of γH_2_AX lesions. On the other hand, when NUDT5 is inhibited or lost, the oxidative stress on the DNA becomes unopposed, resulting in the accumulation of 8-oxoG and γH_2_AX lesions within the nucleus. This accumulation subsequently leads to the slowing down of DNA replication and a reduction in cell proliferation. This proposed mechanism underscores the pivotal role of NUDT5 in maintaining DNA integrity and regulating the growth of TNBC cells by mitigating oxidative DNA damage, and thereby highlights the potential therapeutic value of targeting NUDT5 in patients with TNBC.

Previous studies have emphasized the role of NUDT5 in hormone receptor-positive breast cancers. In response to progesterone stimulation, Wright et al. showed that NUDT5 forms an ATP-dependent chromatin remodeling complex in the ER-positive breast cancer cell nucleus. NUDT5 converts ADPR to ATP, thus generating energy for chromatin remodeling. They demonstrated that NUDT5 was dephosphorylated at threonine 45 upon hormone exposure, resulting in a conformational change in the homodimeric structure and enzymatic activation to convert pyrophosphated-ADPR into ATP [15]. A follow-up study by Pickup et al. [16] demonstrated that breast cancer stem cells require the ATP-generating NUDT5 function to maintain cancer stemness. A recent study by Qi et al. [35] emphasized the importance of NUDT5’s ATP-producing role at DNA damage sites. To evaluate the possibility of utilizing NUDT5 as a prognostic factor, Zhang et al. [36] found that NUDT5 is highly expressed in breast cancer specimens, and patients with high NUDT5 expression have worse clinical outcomes. NUDT5 was also found to be associated with patient prognosis in esophagus, lung and prostate cancer, as demonstrated by Wang et al. [17], Li et al. [18] and Li et al. [19].

Our studies show that NUDT5 depletion in TNBC cells induces DNA oxidative stress and the accumulation of DNA damage lesions including 8-oxoG and γH2AX lesions. Other investigators studying NUDT5 in *C. elegans* have shown that NUDT5 hydrolyzes 8-oxo-dGDP into 8-oxo-GMP, reducing the incorporation of this mutagenic nucleotide into DNA [37]. Previous studies have also demonstrated that NUDT5 suppresses oxidation-induced DNA mutations and that the knockdown of NUDT5 causes increased mutagenesis [38].

The discovery of NUDT5's significant role in breast cancer biology prompted the exploration of small molecule inhibitors targeting NUDT5. Page et al. [33] conducted a comprehensive screening for such inhibitors using the cellular thermal shift assay, ultimately identifying TH5427 as the lead inhibitory compound. In both in vitro and in vivo settings, our investigations revealed that the NUDT5 inhibitor significantly suppresses TNBC tumor growth. After NUDT5 loss, we observed the induction of DNA damage in TNBCs, characterized by the accumulation of γH_2_AX and 8-oxoG lesions within the DNA. This observation understandably raised concerns about potential genotoxic side effects. Our data suggests that these DNA damage lesions are induced after NUDT5 loss primarily in NUDT5-high and ROS-high TNBCs, with minimal impact on tissues characterized by low ROS levels.

To address the pitfalls of the current study, future investigations could explore the chromatin compaction, histone modifications associated with compaction, and replication velocity resulting from impaired replication fork progression in cells depleted of NUDT5. Future endeavors could also delve into the cellular localization of NUDT5 and track the cellular localization of NUDT5 after oxidative stress challenge. More importantly, the successful translation of NUDT5 inhibitors from basic science research into clinical trials hinges on the development of next-generation NUDT5 inhibitors that are both more potent and less toxic.

## Conclusions

The ultimate goal of these studies is to identify critical phosphatases that play a pivotal role in regulating TNBC growth and survival, and evaluate them as potential therapeutic targets. In this context, we have identified NUDT5 as a key protector against DNA oxidative stress in TNBCs. Our findings suggest that tumors characterized by high levels of oxidative stress and elevated NUDT5 expression might exhibit increased sensitivity to NUDT5 inhibitors. Thus, NUDT5 inhibitors hold promise as a potential treatment approach for tumors with high oxidative stress, such as TNBCs, either as single agent treatments or in combination with other anti-cancer therapies. These results serve as a foundation for developing NUDT5 inhibitors for the treatment of aggressive breast cancers. Moreover, this work underscores the significance of phosphatases as promising targets for cancer therapy.

### Supplementary Information

**Additional file 1**. **Figure S1. Overexpressed phosphatases.** Overexpressed phosphatases (TNBC versus normal breast, TNBC versus ER−/HER2+, TNBC versus ER-positive, and TNBC versus non-TNBC) in four publicly available datasets.

**Additional file 2**. **Figure S2. Additional growth assays in TNBC.** (**A**) The efficiency of siRNA knockdown in Fig. 2A is demonstrated by Western blot and qPCR data from samples collected on day 1 (shown in this Supplementary Figure) and day 7 (also shown in Fig. 2). Shown are the results of one experiment for each cell line. This experiment was repeated and the results showed similar siRNA knockdown and TNBC growth suppression results. (**B**) MDA-MB-436 NUDT5 ORF cDNA overexpressing cells treated with siRNA targeting the 3’UTR region of NUDT5 mRNA is shown. siRNA knockdown efficiency is shown by Western blot analysis. The significant differences between day 7 cell counts were determined using Student *t* test (ns, not significant; *, *p* < 0.05; **, *p* < 0.01; ***, *p* < 0.001; ****, *p* < 0.0001).

**Additional file 3**. **Figure S3. IC**_**50**_** of TH5427 in breast cancer cell lines.** (**A**) The TH5427 dose–response curve of various cell lines, including immortalized non-tumorigenic normal-like breast cell lines (MCF-10A, MCF-12A), ER-positive breast cancer cell lines (MCF-7, MDA-MB-361, T-47D, ZR-75-1), and triple-negative breast cancer cell lines (MDA-MB-231, MDA-MB-436, and MDA-MB-468, BT-20). (**B**) A summary of the IC_50_ values for immortalized, non-tumorigenic normal-like breast cell lines, ER-positive breast cancer cell lines and triple-negative breast cancer cell lines. The significant differences of IC_50_ between different groups were determined using by one-way ANOVA (ns, not significant; *, *p* < 0.05). (**C**) Additional IHC images from 4 other MDAMB231 xenograft tumors (2 additional tumors from vehicle-treated mice and 2 additional tumors from TH5427-treated mice) are shown. (**D**) NUDT5 IHC staining for 3 vehicle-treated and 3 TH5427-treated tumors from MDAMB231 xenografts, with additional negative (ER-positive MDA-MB-361) and positive (TNBC MDA-MB-231) cell line controls are shown.

**Additional file 4**. **Figure S4. Annexin V-PI apoptosis assay in MCF7 cells.** FACS analysis of MCF-7 cells treated with DMSO, 10 µM TH5427, siLuc, siNUDT5, and the positive control 10 µM staurosporine. Cells were stained with both Annexin V and PI to detect apoptotic cell populations. Each treatment was conducted in triplicate and has been graphed in Fig. 4C.

**Additional file 5**. **Figure S5. Annexin V-PI apoptosis assay in MDA-MB-231 cells** (**B**) FACS analysis of MDA-MB-231 cells treated with DMSO, 10 µM TH5427, siLuc, siNUDT5, and the positive control 10 µM staurosporine. Cells were stained with Annexin V and PI to detect apoptotic cell populations (both early and late apoptosis). Each treatment was conducted in triplicate and has been graphed in Fig. 4C.

**Additional file 6**. **Figure S6. ROS level after H**_**2**_**O**_**2**_** induction.** The levels of ROS in the ER-positive cell lines ZR-75-1 and MDA-MB-361, and in the TNBC cell lines MDA-MB-231 and MDA-MB-468 were assessed using the ROS-Glo™ H_2_O_2_ assay under basal conditions and following treatment with 50 µM H_2_O_2_. *P*-values are as indicated in the figure.

**Additional file 7**. **Figure S7. Loss of NUDT5 induces oxidative 8-oxoG response.** (**A**) 8-oxoG lesions were stained in TNBC (MDA-MB-436, MDA-MB-468) and ER-positive (ZR-75-1 and MDA-MB-361) cells treated with siLuc or siNUDT5, and nuclei were counterstained with DAPI after 4 days. The data is shown as nuclear intensity for siLuc- or siNUDT5-treated cells. Statistical significance was analyzed by the Student’s *t-*test. Additional TNBC (MDA-MB-231) and ER-positive (MCF-7) cell lines are shown in Fig. 5. Proof of effective knockdown is shown via Western blot and qPCR in **Supplementary Fig. 2A**.

**Additional file 8**. **Figure S8. Loss of NUDT5 induces oxidative 8-oxoG and DNA damage response.** γH_2_AX was stained in TNBC (MDA-MB-436, MDA-MB-468) and ER-positive (ZR-75-1 and MDA-MB-361) cells treated with siLuc or siNUDT5, and nuclei were counterstained with DAPI after 7 days. The data is shown as γH_2_AX positivity, and was compared between the different treatments. Statistical significance was analyzed by the Student’s *t *test. Additional TNBC (MDA-MB-231) and ER-positive (MCF-7) cell lines are shown in Fig. 5. Proof of effective knockdown is shown via Western blot and qPCR in **Supplementary Fig. 2A**.

**Additional file 9**. **Table S1. Breast cancer survival analysis of overexpressed phosphatases.** Survival studies of NUDT5, CDC25A, CDC25B, DLGAP5, IMPA2, and PTPLA in METABRIC [22, 23], Esserman [27], Kao [28], and Pawitan [29] data sets.

## Data Availability

Data and materials are available upon request. Please contact PHBrown@mdanderson.org to access any data or material.

## Abbreviations

ADPR: Adenosine 5′diphosphoribose
BrdU: Bromodeoxyuridine
CIdU: 5-Chloro-2′-deoxyuridine
DAPK1: Death-associated protein kinase 1
ER: Estrogen receptor
H_2_O_2_: Hydrogen peroxide
IC_50_: Half maximal inhibitory concentration
IdU: 5-Iodo-2'-Deoxyuridine
METABRIC: Molecular Taxonomy of Breast Cancer International Consortium
NUDIX: Nucleoside diphosphates linked to moiety-X
NUDT5: Nudix hydrolase 5
PTP: Protein tyrosine phosphatases
pTRIPZ-NUDT5: Inducible lentiviral shRNA of NUDT5
TCGA: The Cancer Genome Atlas
TNBC: Triple-negative breast cancer
XTG9-RO: X-treme-Gene9 transfection agent
