# Correction: The novel phosphatase NUDT5 is a critical regulator of triple-negative breast cancer growth

**DOI:** 10.1186/s13058-024-01814-9

**Published:** 2024-03-26

**Authors:** Jing Qian, Yanxia Ma, William M. Tahaney, Cassandra L. Moyer, Amanda Lanier, Jamal Hill, Darian Coleman, Negar Koupaei, Susan G. Hilsenbeck, Michelle I. Savage, Brent D. G. Page, Abhijit Mazumdar, Powel H. Brown

**Affiliations:** 1 Department of Clinical Cancer Prevention, The University of Texas MD Anderson Cancer Center, Houston, TX USA; 2 Department of Molecular and Cellular Biology, Baylor College of Medicine, Houston, TX USA; 3 Lester and Sue Smith Breast Center and Dan L. Duncan Cancer Center, Baylor College of Medicine, Houston, TX USA; 4 Department of Breast Surgical Oncology, The University of Texas MD Anderson Cancer Center, Houston, TX USA; 5 Faculty of Pharmaceutical Sciences, University of British Columbia, Vancouver, Canada; 6 Monte Rosa Therapeutics, Boston, USA; 7 Department of Neurosurgery, The University of Texas MD Anderson Cancer Center, Houston, TX USA; 8 The University of Texas MD Anderson Cancer Center UTHealth Houston Graduate School of Biomedical Sciences, Houston, TX USA

**Qian et al. Breast Cancer Research (2024) 26:23**

10.1186/s13058-024-01778-w

The original article has incomplete funding information with regard to author Negar Koupaei. The complete funding note is as follows:

**Funding**

This work was funded by a Susan G Komen Promise Grant (KG081694 P.H.B.), a Komen SAB Grant (9SAB12-00006, P.H.B.), and a CCSG Grant (P30 CA016672, P.H.B.). This work was also supported by the John Charles Cain Endowment (P.H.B.). Negar Koupaei (NK) was supported by the 1R25CA240137-01A1 UPWARDS Training Program and by the CPRIT Research Training Award CPRIT Training Program (RP210028).

The original article [1] has been corrected.
