# Supplementary figures and images for: The novel phosphatase NUDT5 is a critical regulator of triple-negative breast cancer growth
Source: Breast Cancer Res. 2024 Feb 5;26:23. doi: 10.1186/s13058-024-01778-w (PMC10845800; doi:10.1186/s13058-024-01778-w)

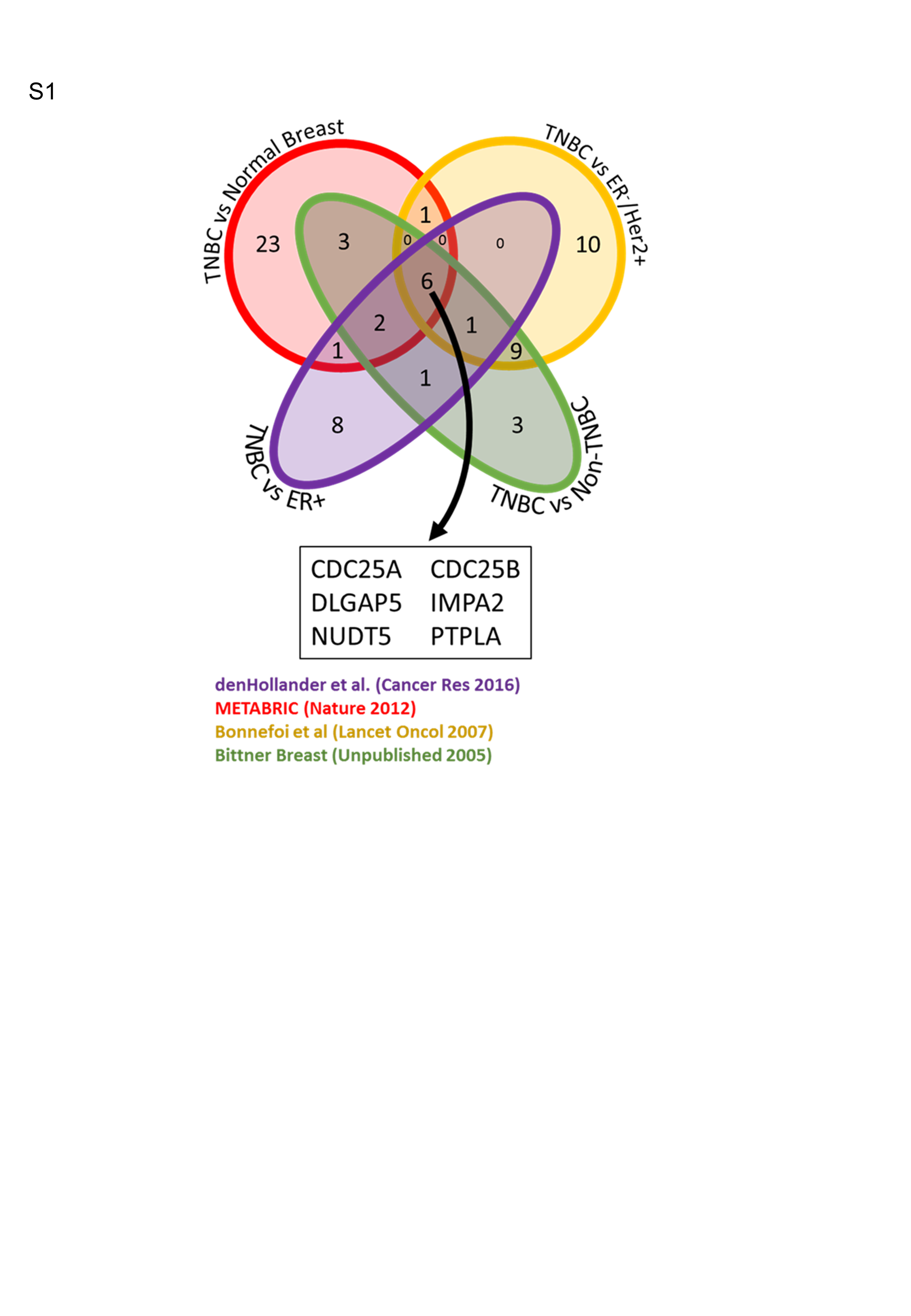

Supplement: Supplementary file 1 — Additional file 1. Figure S1. Overexpressed phosphatases. Overexpressed phosphatases (TNBC versus normal breast, TNBC versus ER−/HER2+, TNBC versus ER-positive, and TNBC versus non-TNBC) in four publicly available datasets. [file 13058_2024_1778_MOESM1_ESM.tif]

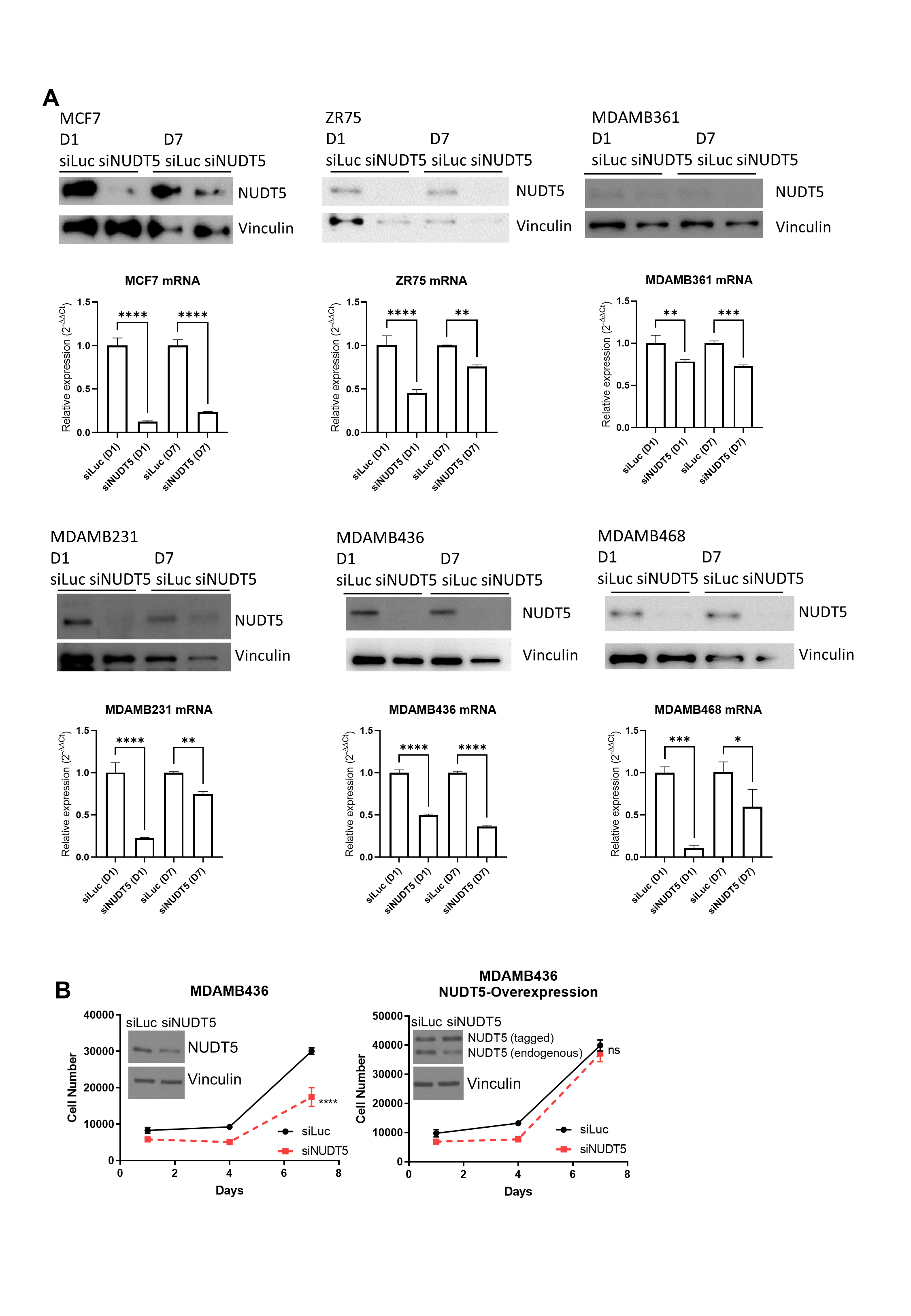

Supplement: Supplementary file 2 — Additional file 2. Figure S2. Additional growth assays in TNBC. (A) The efficiency of siRNA knockdown in Fig. 2A is demonstrated by Western blot and qPCR data from samples collected on day 1 (shown in this Supplementary Figure) and day 7 (also shown in Fig. 2). Shown are the results of one experiment for each cell line. This experiment was repeated and the results showed similar siRNA knockdown and TNBC growth suppression results. (B) MDA-MB-436 NUDT5 ORF cDNA overexpressing cells treated with siRNA targeting the 3’UTR region of NUDT5 mRNA is shown. siRNA knockdown efficiency is shown by Western blot analysis. The significant differences between day 7 cell counts were determined using Student t test (ns, not significant; *, p < 0.05; **, p < 0.01; ***, p < 0.001; ****, p < 0.0001). [file 13058_2024_1778_MOESM2_ESM.tif]

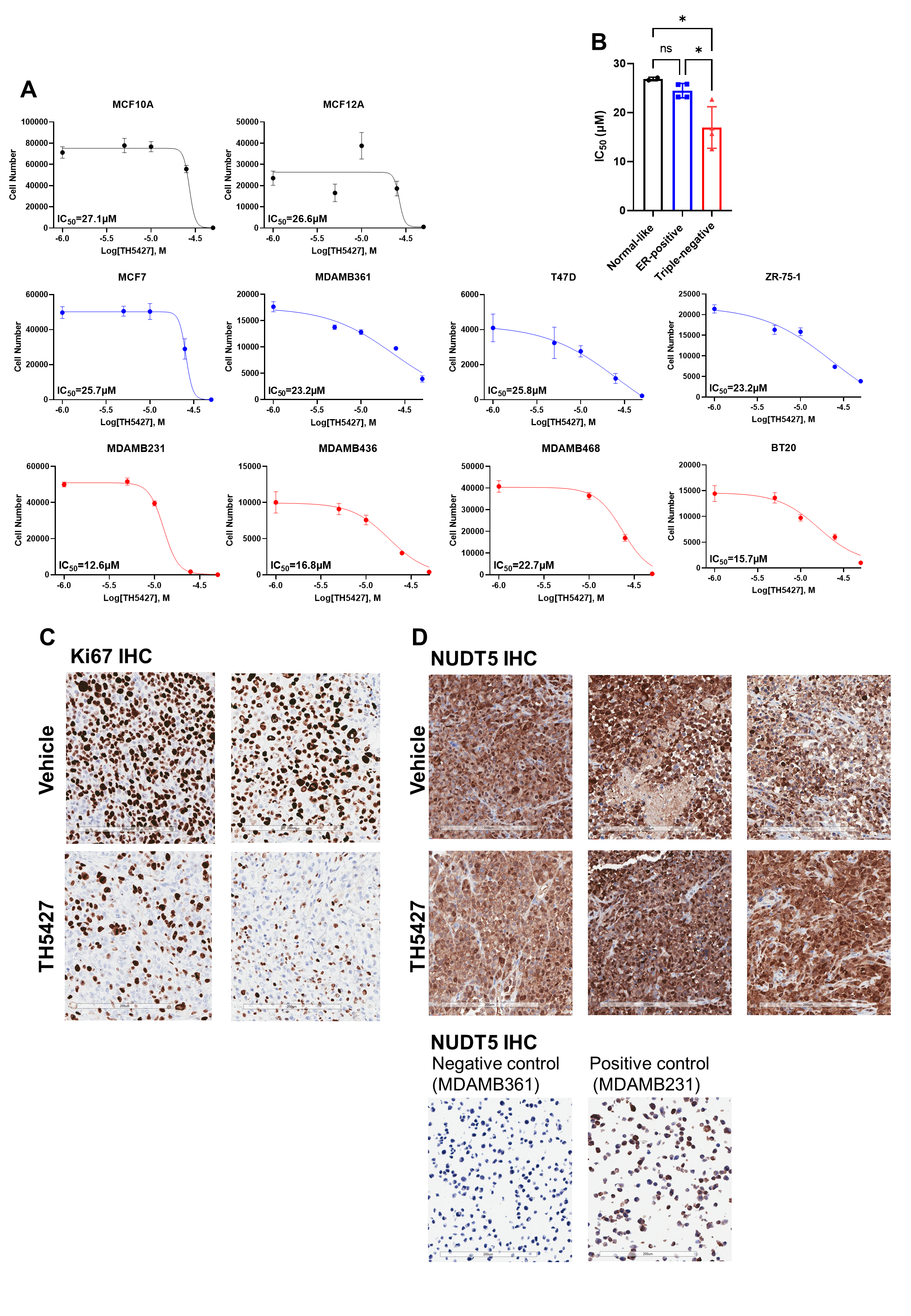

Supplement: Supplementary file 3 — Additional file 3. Figure S3. IC50 of TH5427 in breast cancer cell lines. (A) The TH5427 dose–response curve of various cell lines, including immortalized non-tumorigenic normal-like breast cell lines (MCF-10A, MCF-12A), ER-positive breast cancer cell lines (MCF-7, MDA-MB-361, T-47D, ZR-75-1), and triple-negative breast cancer cell lines (MDA-MB-231, MDA-MB-436, and MDA-MB-468, BT-20). (B) A summary of the IC50 values for immortalized, non-tumorigenic normal-like breast cell lines, ER-positive breast cancer cell lines and triple-negative breast cancer cell lines. The significant differences of IC50 between different groups were determined using by one-way ANOVA (ns, not significant; *, p < 0.05). (C) Additional IHC images from 4 other MDAMB231 xenograft tumors (2 additional tumors from vehicle-treated mice and 2 additional tumors from TH5427-treated mice) are shown. (D) NUDT5 IHC staining for 3 vehicle-treated and 3 TH5427-treated tumors from MDAMB231 xenografts, with additional negative (ER-positive MDA-MB-361) and positive (TNBC MDA-MB-231) cell line controls are shown. [file 13058_2024_1778_MOESM3_ESM.tif]

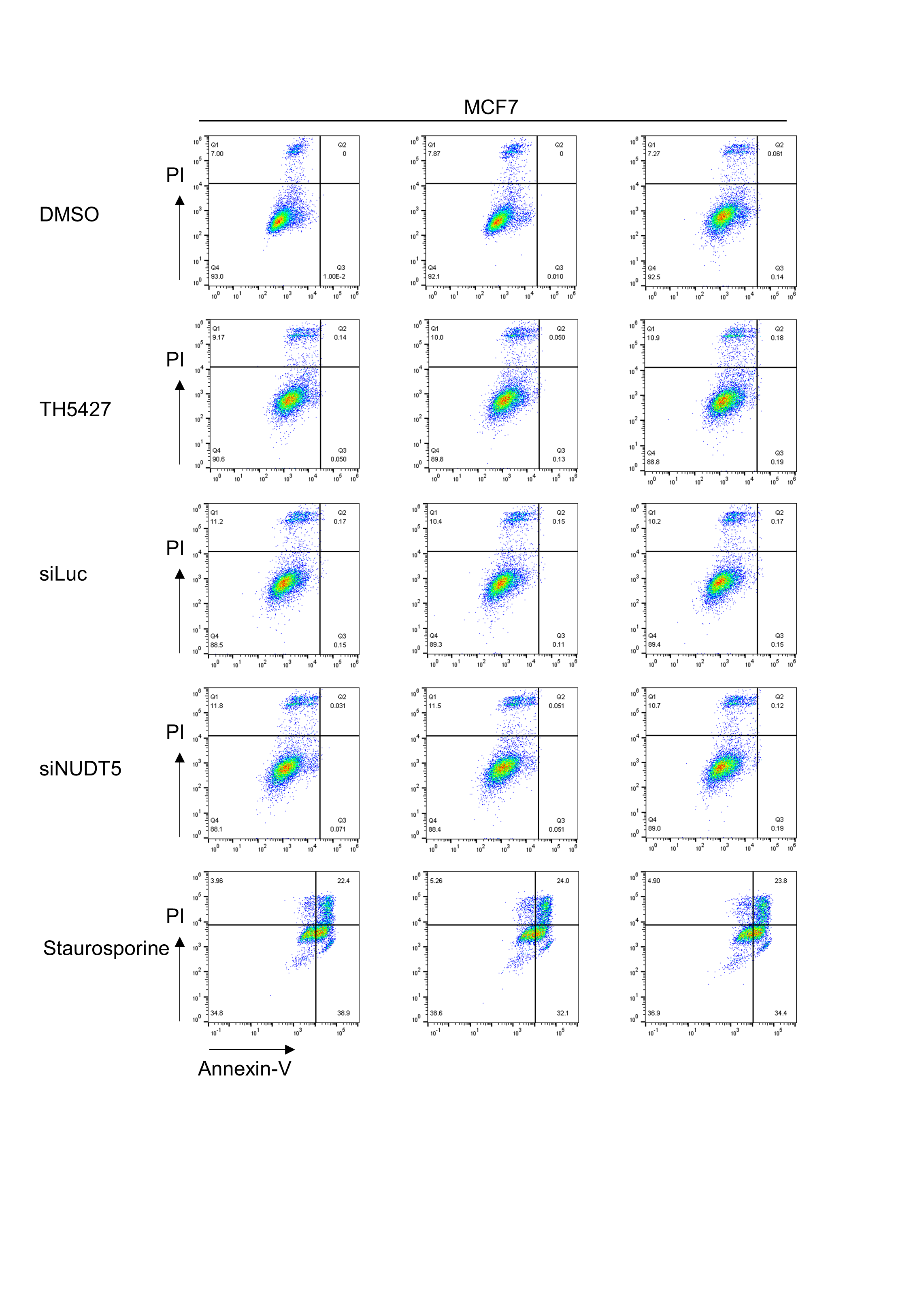

Supplement: Supplementary file 4 — Additional file 4. Figure S4. Annexin V-PI apoptosis assay in MCF7 cells. FACS analysis of MCF-7 cells treated with DMSO, 10 µM TH5427, siLuc, siNUDT5, and the positive control 10 µM staurosporine. Cells were stained with both Annexin V and PI to detect apoptotic cell populations. Each treatment was conducted in triplicate and has been graphed in Fig. 4C. [file 13058_2024_1778_MOESM4_ESM.tif]

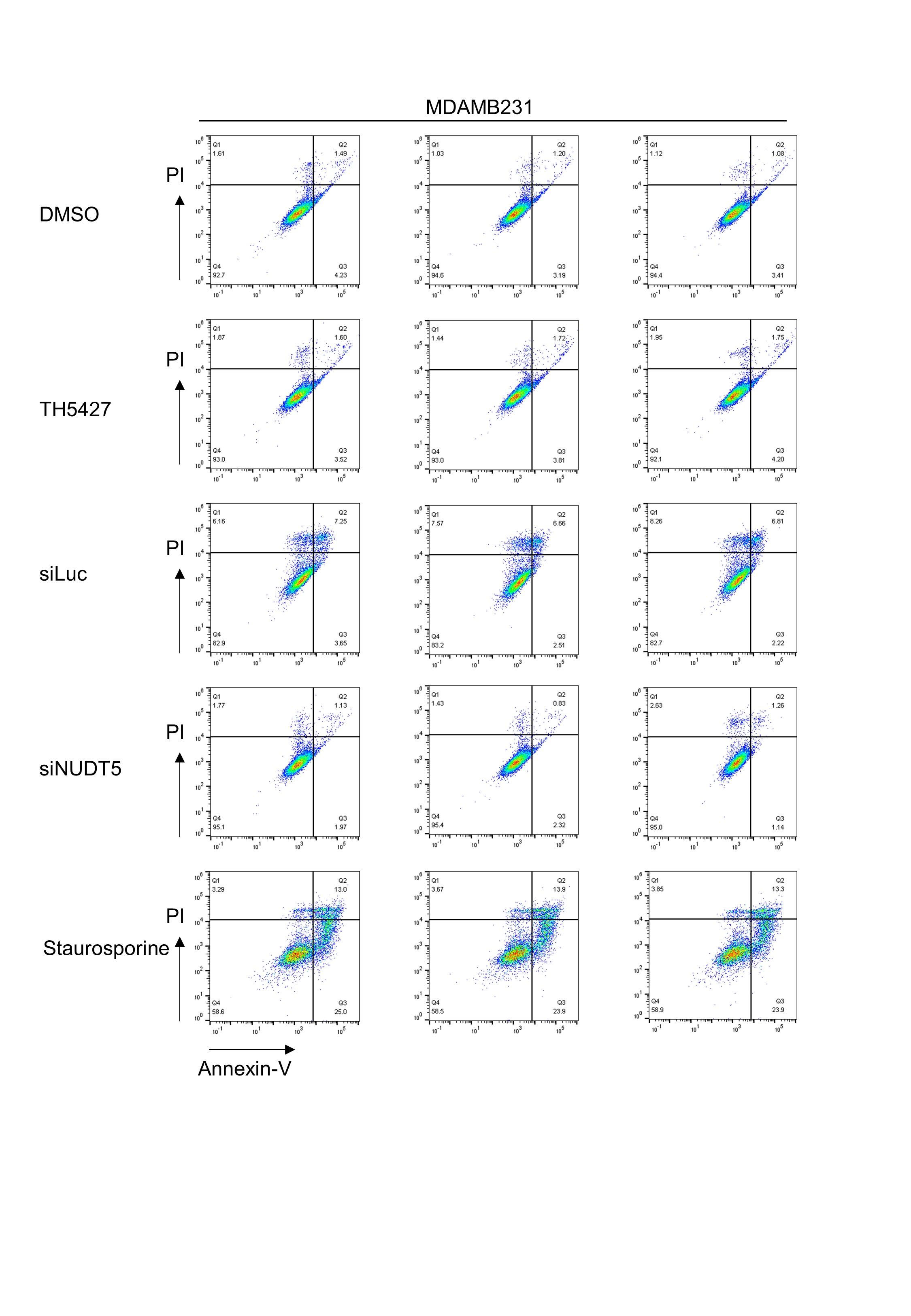

Supplement: Supplementary file 5 — Additional file 5. Figure S5. Annexin V-PI apoptosis assay in MDA-MB-231 cells (B) FACS analysis of MDA-MB-231 cells treated with DMSO, 10 µM TH5427, siLuc, siNUDT5, and the positive control 10 µM staurosporine. Cells were stained with Annexin V and PI to detect apoptotic cell populations (both early and late apoptosis). Each treatment was conducted in triplicate and has been graphed in Fig. 4C. [file 13058_2024_1778_MOESM5_ESM.tif]

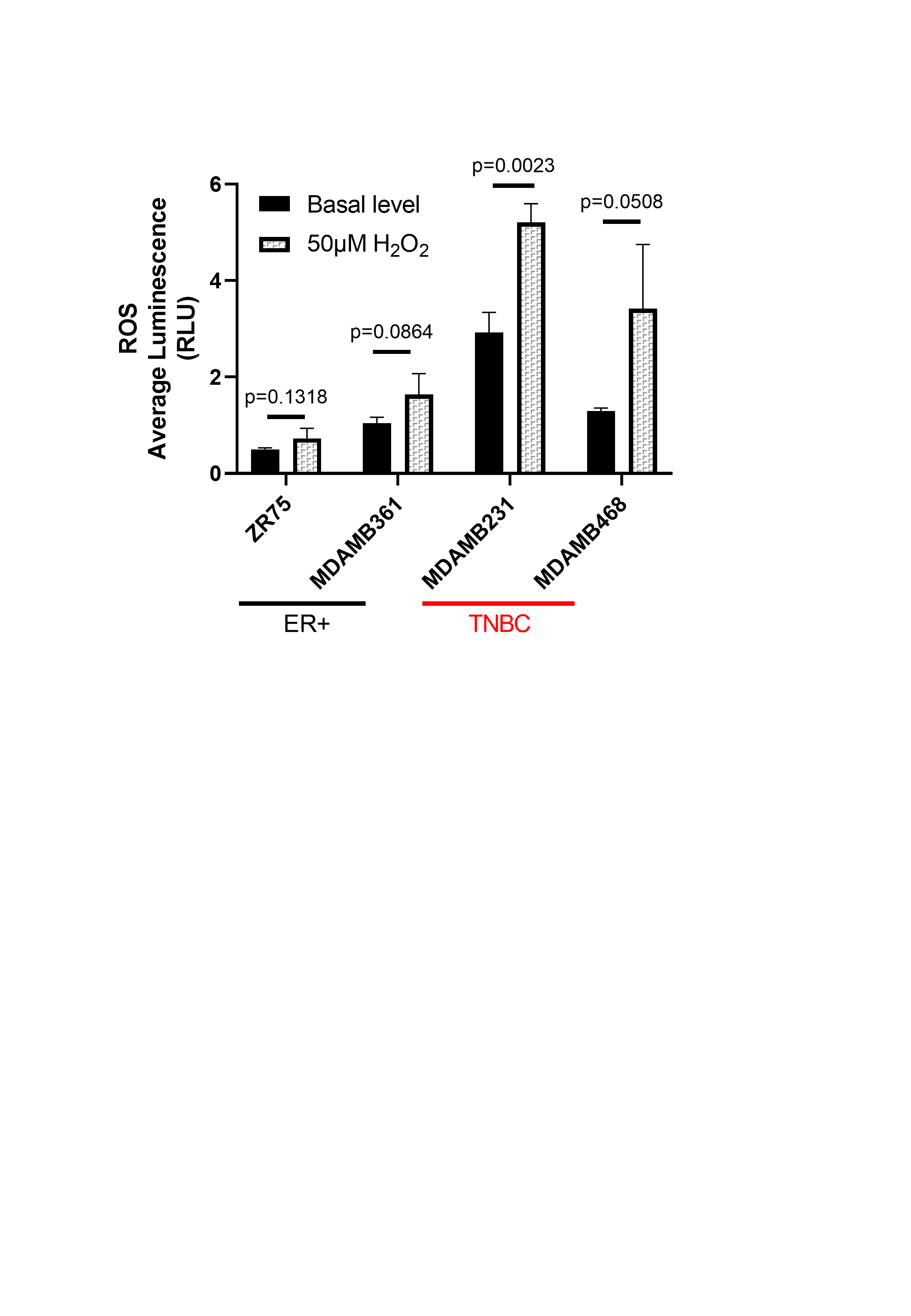

Supplement: Supplementary file 6 — Additional file 6. Figure S6. ROS level after H2O2 induction. The levels of ROS in the ER-positive cell lines ZR-75-1 and MDA-MB-361, and in the TNBC cell lines MDA-MB-231 and MDA-MB-468 were assessed using the ROS-Glo™ H2O2 assay under basal conditions and following treatment with 50 µM H2O2. P-values are as indicated in the figure. [file 13058_2024_1778_MOESM6_ESM.tif]

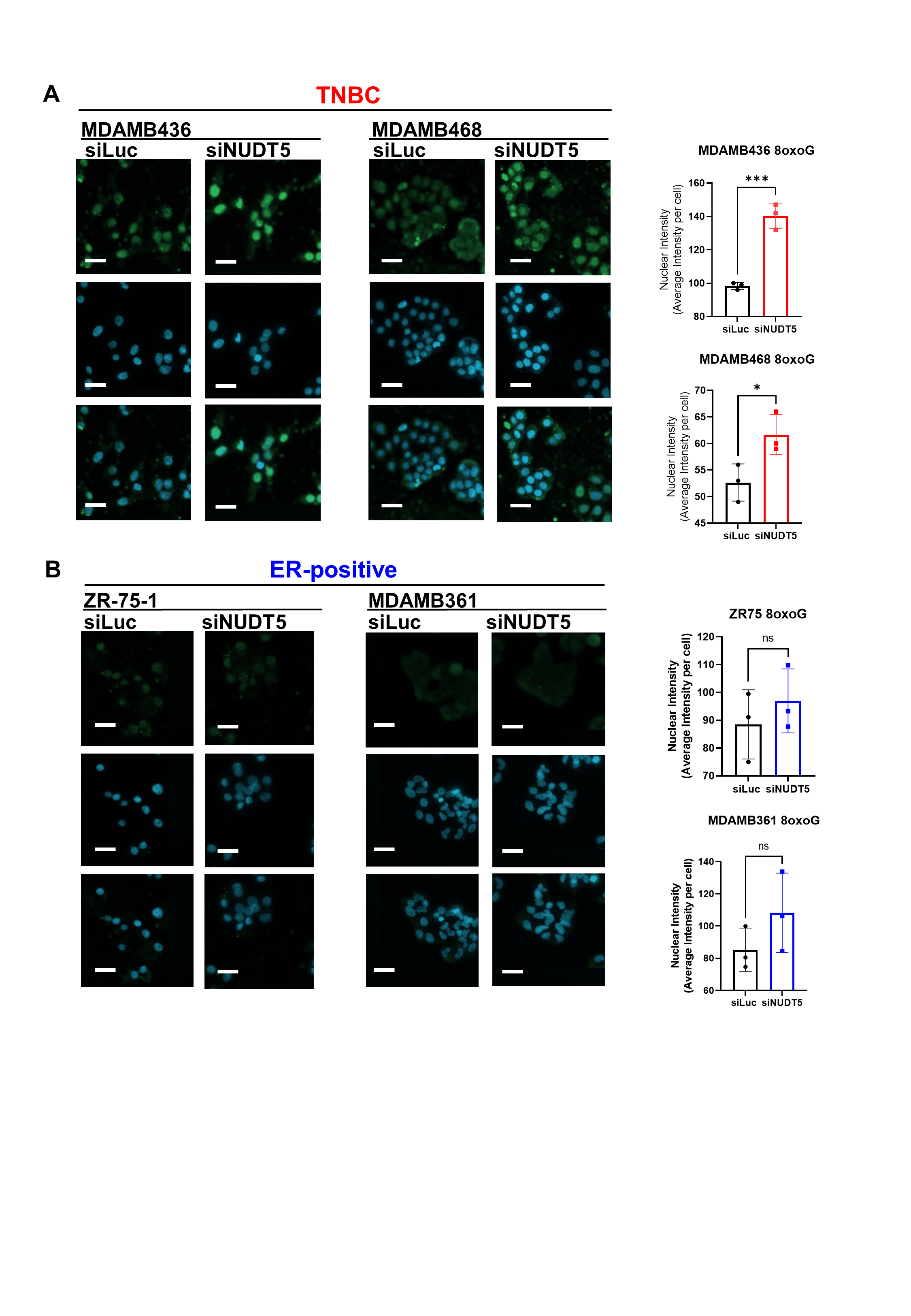

Supplement: Supplementary file 7 — Additional file 7. Figure S7. Loss of NUDT5 induces oxidative 8-oxoG response. (A) 8-oxoG lesions were stained in TNBC (MDA-MB-436, MDA-MB-468) and ER-positive (ZR-75-1 and MDA-MB-361) cells treated with siLuc or siNUDT5, and nuclei were counterstained with DAPI after 4 days. The data is shown as nuclear intensity for siLuc- or siNUDT5-treated cells. Statistical significance was analyzed by the Student’s t-test. Additional TNBC (MDA-MB-231) and ER-positive (MCF-7) cell lines are shown in Fig. 5. Proof of effective knockdown is shown via Western blot and qPCR in Supplementary Fig. 2A. [file 13058_2024_1778_MOESM7_ESM.tif]

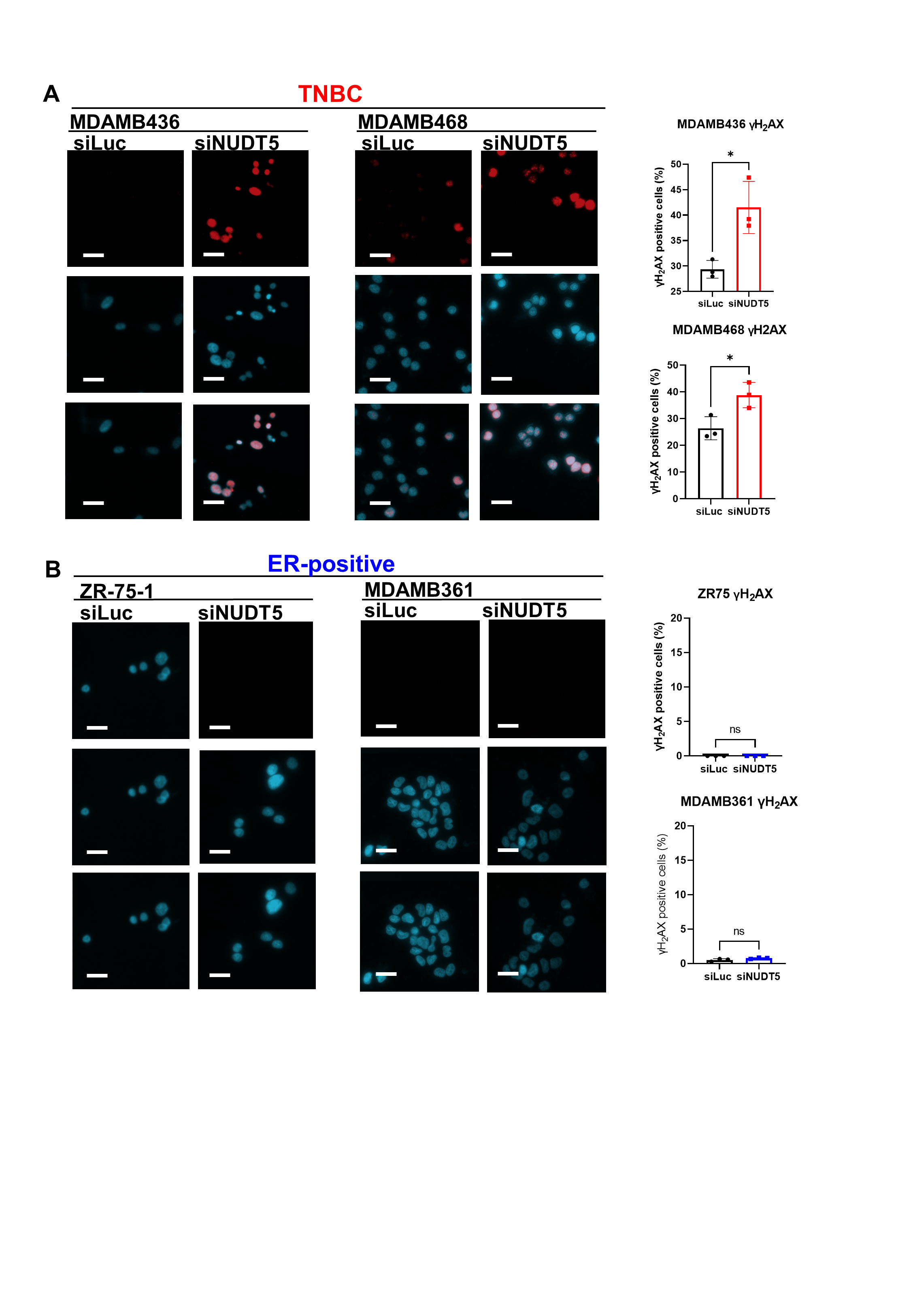

Supplement: Supplementary file 8 — Additional file 8. Figure S8. Loss of NUDT5 induces oxidative 8-oxoG and DNA damage response. γH2AX was stained in TNBC (MDA-MB-436, MDA-MB-468) and ER-positive (ZR-75-1 and MDA-MB-361) cells treated with siLuc or siNUDT5, and nuclei were counterstained with DAPI after 7 days. The data is shown as γH2AX positivity, and was compared between the different treatments. Statistical significance was analyzed by the Student’s t test. Additional TNBC (MDA-MB-231) and ER-positive (MCF-7) cell lines are shown in Fig. 5. Proof of effective knockdown is shown via Western blot and qPCR in Supplementary Fig. 2A. [file 13058_2024_1778_MOESM8_ESM.tif]

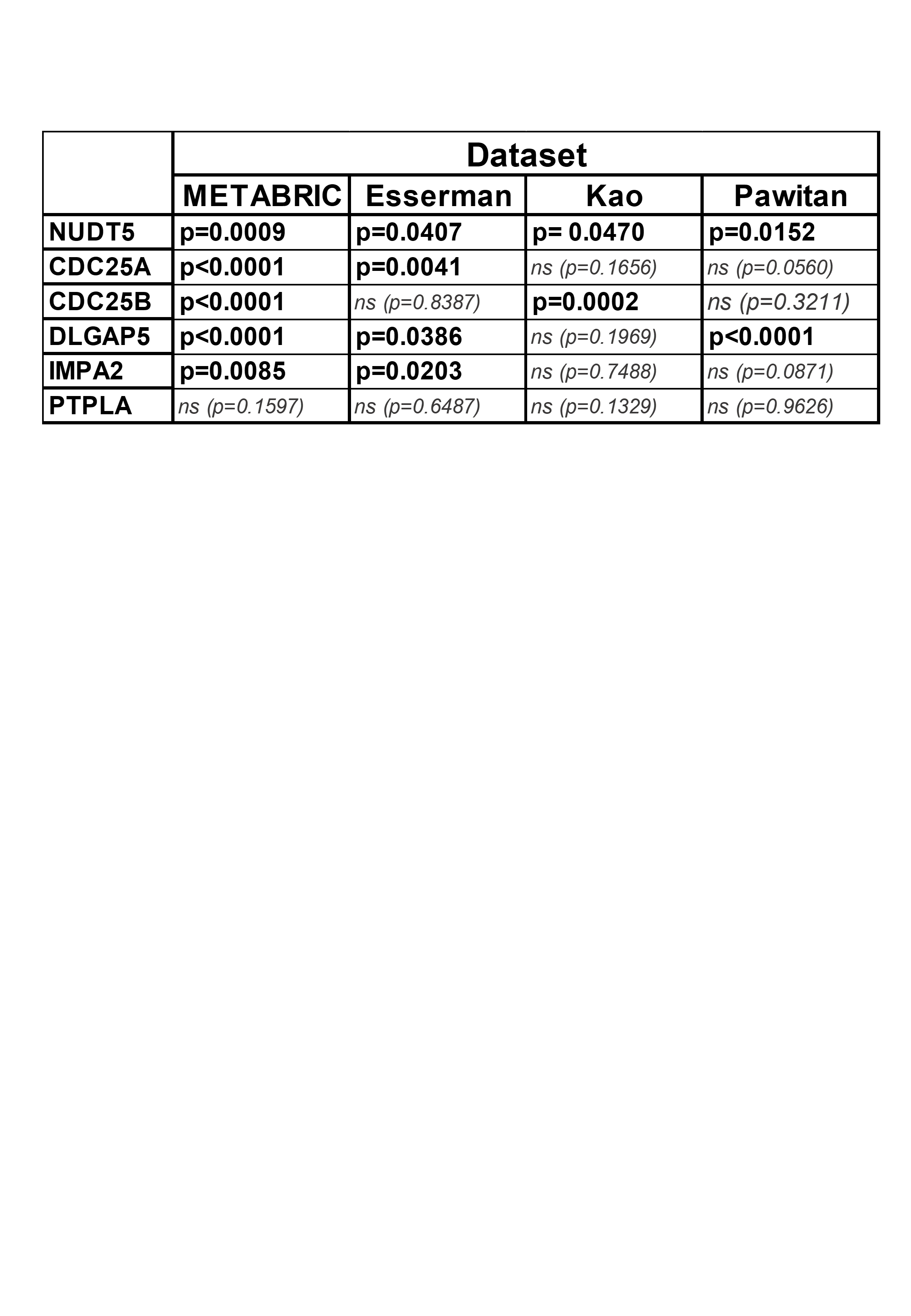

Supplement: Supplementary file 9 — Additional file 9. Table S1. Breast cancer survival analysis of overexpressed phosphatases. Survival studies of NUDT5, CDC25A, CDC25B, DLGAP5, IMPA2, and PTPLA in METABRIC [22, 23], Esserman [27], Kao [28], and Pawitan [29] data sets. [file 13058_2024_1778_MOESM9_ESM.tif]
